# Systemic Approaches for Emission Reduction in Industrial Plants Based on Physical Accounting: Example for an Aluminum Smelter

**DOI:** 10.1021/acs.est.1c05681

**Published:** 2022-01-19

**Authors:** Romain G. Billy, Louis Monnier, Even Nybakke, Morten Isaksen, Daniel B. Müller

**Affiliations:** † Industrial Ecology Programme, Department of Energy and Process Engineering, Norwegian University of Science and Technology (NTNU), Høgskoleringen 5, 7034 Trondheim, Norway; ‡ Utopies, 25 Rue Titon, 75011 Paris, France; § Hydro Aluminium, Drammensveien 264, 0283 Oslo, Norway

**Keywords:** material flow analysis, carbon accounting, aluminum smelting, material accounting, material and energy efficiency, systems analysis

## Abstract

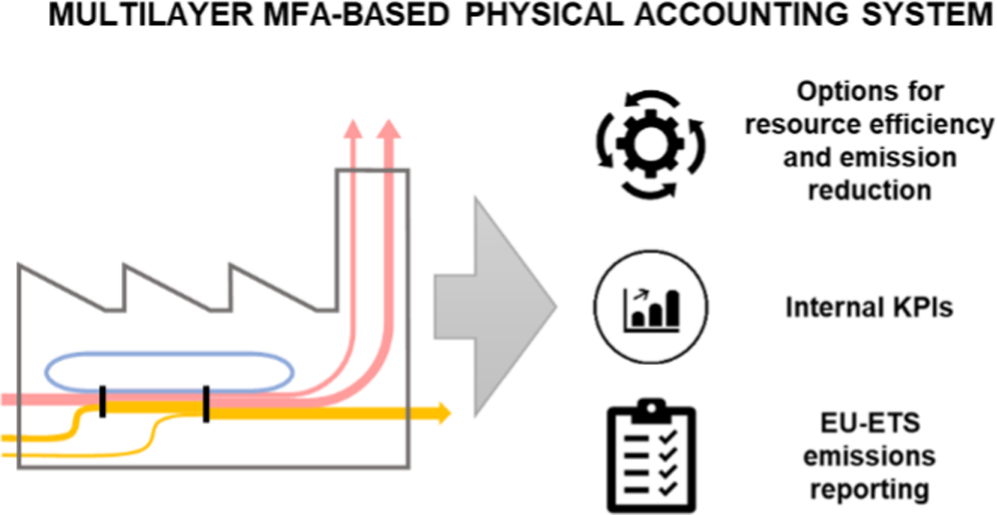

Greenhouse gas (GHG) accounting in industrial plants usually has multiple purposes, including mandatory reporting, shareholder and stakeholder communication, developing key performance indicators (KPIs), or informing cost-effective mitigation options. Current carbon accounting systems, such as the one required by the European Union Emission Trading Scheme (EU ETS), ignore the system context in which emissions occur. This hampers the identification and evaluation of comprehensive mitigation strategies considering linkages between materials, energy, and emissions. Here, we propose a carbon accounting method based on multilevel material flow analysis (MFA), which aims at addressing this gap. Using a Norwegian primary aluminum production plant as an example, we analyzed the material stocks and flows within this plant for total mass flows of goods as well as substances such as aluminum and carbon. The results show that the MFA-based accounting (i) is more robust than conventional tools due to mass balance consistency and higher granularity, (ii) allows monitoring the performance of the company and defines meaningful KPIs, (iii) can be used as a basis for the EU ETS reporting and linked to internal reporting, (iv) enables the identification and evaluation of systemic solutions and resource efficiency strategies for reducing emissions, and (v) has the potential to save costs.

## Abstract 2

Taking the example of an aluminum smelter, we show how physical accounting based on plant-level material flow analysis has the potential to improve carbon accounting schemes and inform mitigation strategies through a systemic understanding of the linkages between materials, energy, and emissions.

## Introduction

The industry sector contributed just over 30% of the global greenhouse gas (GHG) emissions in 2010,^1^ and the aluminum value chain alone embodied in 2009 approximately 1.1% of the global GHG emissions, whereof 90% was associated with primary production,^2^ which is expected to keep soaring for decades.^3^ If global warming were to be stabilized at 2 °C above pre-industrial levels, the carbon intensity in the industrial sector would decrease by 60% in 2050 compared to 2010 levels.^4^ Emission trading systems (ETS), such as the European Union ETS (EU ETS), are established to help fulfill this goal.^5^ The EU ETS currently requires industrial installations from 28 sectors (including primary aluminum production) to account for their direct (scope 1) CO_2_, N_2_O, and perfluorinated compound (PFC) emissions, covering 45% of EUʼs total territorial carbon dioxide emissions.^6^ A cap on these emissions was established at the EU level in 2013 (phase 3) and set to decrease by 1.74% each year and by 2.2% from 2021 onward (phase 4).^7^

Emission accounting is a prerequisite to any ETS to spot the biggest contributors, assign responsibility, and track performance evolution over time. Under the EU ETS, this is carried out following guidelines and methodologies issued by the EU,^8^ which aim to standardize the accounting process but not to identify and evaluate emission reduction strategies. Industrial installations are only required to report their total direct GHG emissions, even though they can comprise several technical units with different inputs and outputs. These highly aggregated results have little operational meaning and are unsuitable for comparison, especially since the interpretation of the accounting rules may differ from one site to another.^9^ Moreover, the data can have large uncertainties and may not be mass balance consistent, which is not addressed by the current accounting methodology. This reduces the robustness of the accounting and increases the risk of not detecting errors coming from uncertainties or a poorly defined system (missing flows or stocks). Finally, the EU ETS only covers a limited number of GHGs and does not give credit for improving the end-of-life (EoL) management of waste flows (through better separation, reuse, or recycling).

Tang and Luo^10^ showed that companies with higher quality carbon management systems tend to achieve higher emission reductions but observed that carbon accounting and auditing alone had a limited effect, which they attributed among others to the lack of international standards. Indeed, because of the above-mentioned limitations, the EU ETS accounting is not the best tool to inform decision makers and plant managers about the performance of the sites. As a result, companies often develop and maintain separate accounting frameworks with an aim to inform mitigation strategies. However, these internal corporate accounting frameworks, although more refined, tend to neglect the systemic linkages between carbon emissions, materials, and energy. To understand causalities of emissions not only at the points where emissions occur but also emission changes caused throughout the system due to changes in material flows, carbon should be tracked (i) not only as emissions but throughout the system, in raw material inflows, intermediates, and byproducts, and (ii) not in isolation but understood as part of a complex system with feedbacks and delays. Climate change mitigation decisions based on attributional life cycle assessment frameworks and inventories might then lead to unintended systemic consequences.^11^ In addition, these frameworks often list incomplete information^12^ and are unsuitable for comparison between sites; hence, they are not suited to inform investors,^13^ internal decision makers, and other stakeholders. Meanwhile, monitoring, reporting, and verifying emissions in the EU ETS represent an average yearly cost of 22 000 Euros per installation included. In relation to total emissions, these operational costs alone amount to 0.07 Euros per ton of carbon dioxide emitted^14^ and stand for the greater part of the overall transaction costs associated with participating in the EU ETS.^14−16^ However, despite such costs, there is still a lack of accounting tools that enable companies to identify and evaluate alternative strategies for saving resources and emissions.

Historically, material and energy balances at plant level have been used in steel production systems as part of the flowsheeting approach, originally developed for process optimization.^17−19^ Porzio et al.^19^ developed a decision support system for the steel industry based on flowsheeting, in which they modeled the main flows of products and materials within a plant and linked those flows with carbon dioxide emissions, enabling us to conduct forecasts and scenario analyses. While some early material flow analysis (MFA) studies had a plant-level focus,^20^ this method has been mostly used as a tool to study global, national, or regional material cycles,^21^ and very few plant-level MFAs have been performed so far. This is particularly the case for multilayer MFAs, which trace multiple individual chemical elements. The tracing of individual chemical elements is relevant for controlling the qualities of the main products, byproducts, and wastes or emissions. The optimization of the qualities of the different outputs, in turn, can have significant implications on the energy use and emissions. Plant-level MFA has recently regained attention, specifically to account for GHG emissions of steel production systems,^22,23^ but those studies usually differentiate only one layer (total mass) and do not trace individual chemical elements/substances in designated layers. Some studies extended the spatial boundaries of the analysis beyond a single plant, such as Wu et al.^24^ who analyzed the yearly exergy and energy flows as well as the carbon dioxide emissions of an iron and steel industrial network. Likewise, the scope has been extended to factory buildings, including, for instance, air conditioning and heating.^25,26^ These approaches unveil greater potentials to reduce emissions, yet they differ with the perimeter commonly used to account for GHG emissions in the industry such as in the EU ETS guidelines. Gonzalez Hernandez et al.^27^ used control data (i.e., with a very high temporal resolution) to quantify exergy flows and study resource efficiency in a steel plant. Their results gave operational details regarding the improvement measures that need to be implemented but are not linked with the yearly GHG emissions of the complete plant.

When it comes to aluminum, despite being one of the most studied metal cycles^28^ at the global,^2,29,30^ regional,^31^ and country^32−37^ levels, plant-level applications have been scarce. Hannula et al.^38^ developed a simulation-based flowsheet for aluminum recycling and studied its resource efficiency through exergy analysis and life cycle assessment, but their system definition did not include a real-scale plant. While smelting is the most important process for both direct and indirect emissions in the aluminum cycle,^2^ we could not find previous studies quantifying the entire metabolism of a primary aluminum plant nor did we find applications of MFA-based physical accounting for improving GHG emissions accounting, reporting, and mitigation.

Here, we perform a multilayer MFA to describe in a system context the metabolism of Norsk Hydroʼs primary aluminum smelter in Sunndal, Norway (the largest European smelter excl. Russia, with a design capacity of 300 + 100 kt Al/year in two smelting lines). We use this example to show how accounting tools that regard emissions as part of a larger production system can help to

(i) quantify GHG emissions of industrial facilities based on mass balance consistent physical accounting;
(ii) facilitate the identification of emission mitigation strategies to reach the EU ETS emission reduction targets—such as enhancing resource efficiency, substituting energy carriers, and improving specific processes; and
(iii) identify new levers to improve the sustainability performance of an industrial site by addressing systemic effects beyond the EU ETS scope.

## Methods

### Plant-Level Multilayer Material Flow Analysis

**Figure 1:**
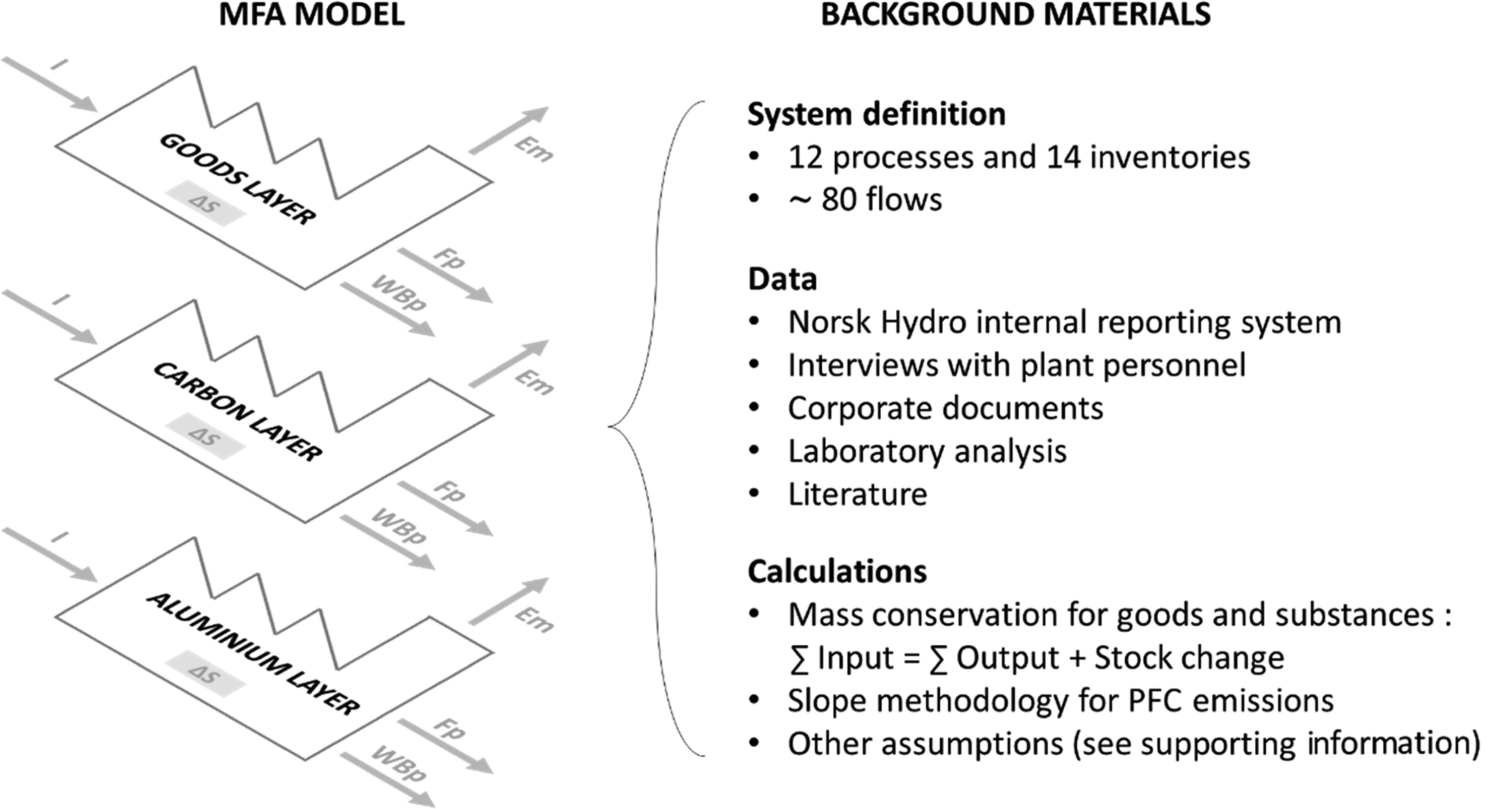
General principle for multilayer MFA model development. *I* = inputs; Δ*S* = stock change; Em = emissions; Fp = final products; WBp = waste and byproducts.

**Figure 2:**
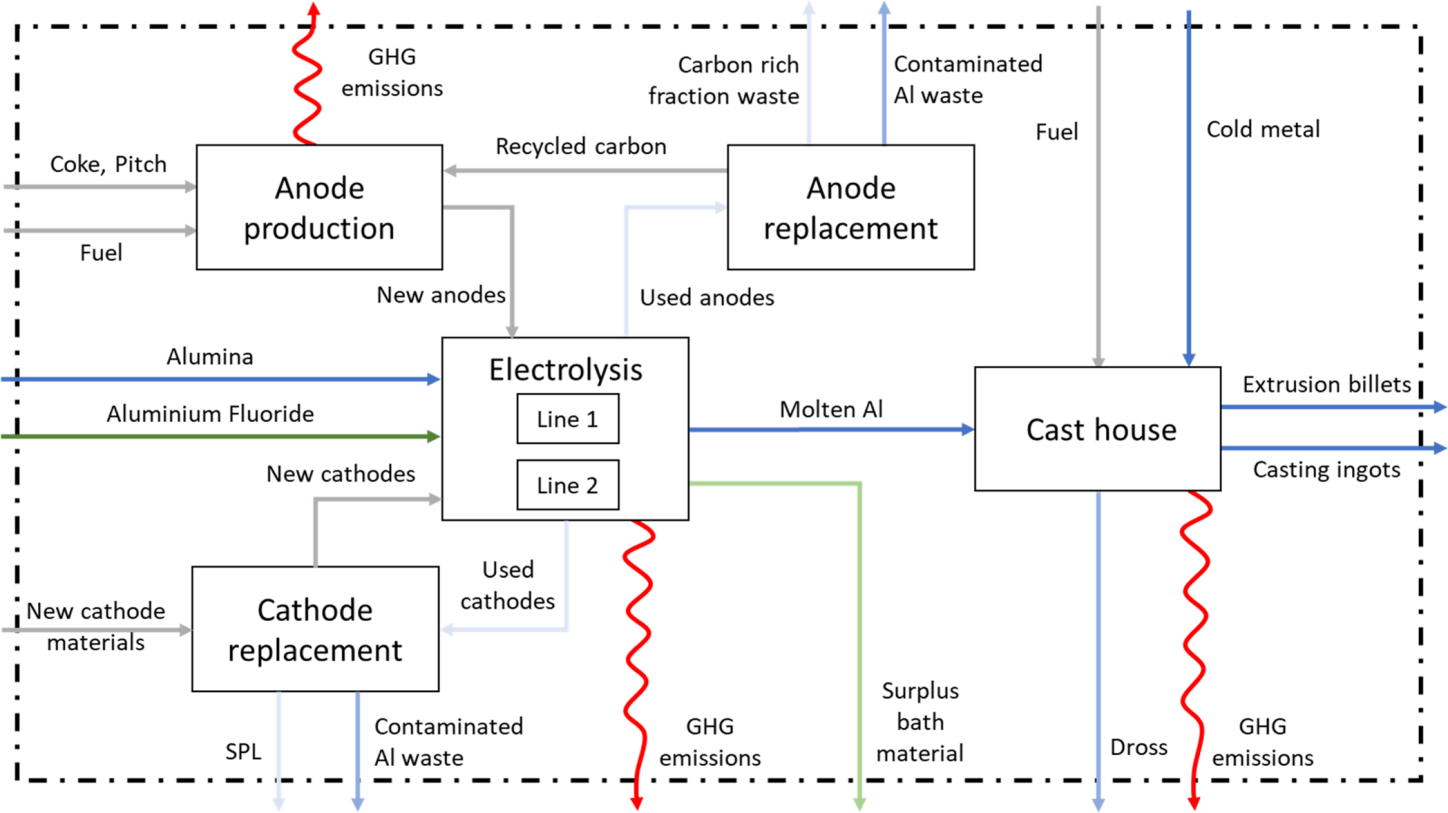
Simplified system definition of the plant. Aluminum-rich flows are shown in blue, carbon-rich flows in gray, fluorine-rich flows in green, and GHG emissions in red. Waste flows are shown in a lighter shade. SPL = spent potlining.

We quantified the metabolism of the plant using the MFA methodology as described by Baccini and Brunner,^39^ which tracks not only goods but also individual substances (a multilayer approach). Our system is quantified for three layers: goods, aluminum, and carbon. Figure 1 summarizes the general principle used in this study to perform the multilayer MFA. Stocks and flows of materials within the plant were quantified first for the goods layer. The aluminum and carbon layers were derived from the goods layer using concentrations of those two elements in the different goods. One of the basic principles of MFA is the conservation of mass, which holds for the three layers. This multilayer physical accounting allows us to better track the fate of individual chemical elements and improve the accuracy of the results by applying element-wise mass balance (Figure 2).

### System Definition

The primary aluminum plant includes two smelting lines, a cast house, and three units dedicated to carbon anodes: one producing them, one rodding them to prepare them for smelting, and one cleaning the used anodes. The system was quantified for the three layers: goods, aluminum, and carbon. To be consistent with the EU ETS scope, the carbon layer covers the whole plant. The goods and the elemental aluminum layers are quantified for the whole plant with the exception of the cast house due to the complexity and limited data availability for the numerous flows of alloying elements.

The plant produces carbon anodes by mixing imported carbon-rich primary materials (tar pitch, petroleum coke) and recycled used anodes (so-called anode butts (AB)) into a paste. The anodes are subsequently shaped, baked, and then attached to a steel rod to be used in the smelting lines, where aluminum oxide is melted in a molten electrolytic bath. Aluminum fluoride and sodium carbonate are added for process control. Rodded carbon anodes are placed on top of the cells, and a carbon cathode is located at the bottom of the cell. While electric current goes from the anodes to the cathode through the molten bath, the carbon contained in the anodes binds with oxygen atoms in aluminum oxide and is emitted to the atmosphere (the Hall–Héroult process). The ideal theoretical reaction emits only CO_2_, yet when the alumina concentration in the electrolytic bath is too low, a phenomenon called the anode effect (AE) occurs during which PFCs are emitted. Additionally, CO can be formed in the pots in a non-neglectable fraction due to the Boudouard reaction^40,41^ and the back reaction.^42^ Although the carbon anodes are covered with anode cover material (ACM), a mixture of bath material (mostly composed of cryolite and chiolite) and alumina, part of it oxidizes with the ambient air.^40^ The liquid aluminum resulting from this process sinks to the bottom of the cells, where it is tapped out daily. The molten aluminum is mixed with alloying elements and solid aluminum metal in the cast house to produce primary foundry alloys or extrusion billets.

The anodes have an average lifetime of 4 weeks, after which the remaining butts are removed from the pots and cleaned in several steps. This generates different waste flows that leave the plant for energy recovery or landfilling. The remaining clean carbon-rich fraction is recycled to make new anodes, either internally or externally. The average lifetime of a pot that contains the cathode is 4–6 years, after which pots are delined and relined with new refractory materials and a cathode. The waste from this process, called spent pot lining (SPL), is sorted into a contaminated carbon-rich fraction (first cut) and a contaminated, used refractory material fraction (second cut).

### Quantification and Data Sources

All three layers were quantified for the year 2017. Inventories were introduced whenever necessary to capture relevant stock changes that might have occurred in the plant during the study year. Moreover, it was assumed that there was no stock change in the smelting lines (i.e., no stock change in the pots used to reduce alumina). The system was quantified using mostly internal reporting data. In cases of lacking or poor data, assumptions and estimates were made based on scientific literature, interviews with plant personnel, and corporate documents, or with the use of the mass balance principle. The carbon-containing exhaust gas from electrolysis was assumed to consist of CO_2_, CO, and PFCs (Section S1). This assumption is consistent with the measured values in similar smelting lines^41^ where other gases have proven to be present in negligible fractions. The ratio of CO_2_ to CO emitted is assumed to be the same as the one measured by Kimmerle et al.^41^ in a similar pot design, although CO emissions are neglected by the EU ETS methodology (Section S2.2). PFC emissions are calculated according to the slope methodology (Section S2.3) also used by the EU ETS^8^ [Annex IV Section 8].

### Potential for Emission Reduction

To illustrate the capabilities of the MFA approach, we used the system definition to identify the most promising technological measures to limit the overall plant-level emissions, making sure that the reduction in one process also minimizes the undesired impacts over the whole system. Table 1 lists those measures and shows how the potential emission reductions were calculated for the different intervention options. Detailed calculations are available in Section S3. All of these measures have been or are currently being considered by the aluminum industry, even if their implementation remains limited by uncertainties regarding economic profitability. The feasibility is not described further both for confidentiality reasons and because the main objective of this study is to demonstrate the potential of MFA for physical accounting, practical implementation of the reduction measures being out of the scope. The replacement of carbon anodes with inert anodes was not considered due to a lack of information about the implementation of this technology, including the feasibility of retrofitting of current smelting plants and potential trade-offs in energy use.^43^ Besides, our current system definition would not be appropriate for a plant using inert anodes: entire subsystems like anode production and anode replacement would become obsolete, while new processes might need to be added, making a direct comparison difficult.

**Table 1:** Emission Reduction Estimation Method for Different Measures

measure	calculation for theoretical direct GHG emission reduction
improving alumina reduction (i.e., reaction occurring inside the pots during smelting)	difference between the calculated GHG produced in the anode gas and the theoretical GHG emissions from alumina reduction following the ideal reaction
reducing air burn	difference between the calculated GHG emissions in the exhaust gas from smelting and the calculated GHG emissions in the anode gas
diminishing AE	calculated PFCs emissions using the EU ETS slope methodology
limiting the amount of excess carbon (nonoxidized anodes) supplied to smelting	calculated assuming that all anodes were produced with the same carbon intensity as the one produced in the studied plant
reducing waste generation during anode production	assuming that the production of useful outputs of each process remains constant, that reducing waste generation allows us to decrease inputs and that the amount of fuel supplied is proportional to the total input of the process, we estimated GHG emission reduction potential for the anode paste plant, the anode baking furnace, and the anode rodding process
replacing liquid natural gas (LNG) in the anode plant and cast house with GHG-free energy carriers (hydrogen or electricity)	GHG emissions from LNG

### Uncertainties and Limitations

Norsk Hydroʼs internal reporting system provided reliable data to quantify most material flows. Nevertheless, some parts of the system were quantified using assumptions with a relatively high uncertainty, such as for the flows related to SPL production or the ratio of CO_2_ to CO emitted to the atmosphere. A qualitative analysis of the level of uncertainty of the main parameters and assumptions as well as quantification methods for the different flows is presented in Sections S4 and S5, Supporting Information.

To understand the potential influence on the results of the most uncertain parameters, a one-factor-at-a-time sensitivity analysis was conducted on the main GHG emission flows (emissions from the smelting lines and the anode baking furnace). A description of the methodology and detailed results are presented in Sections S6 and S7.

## Results

### Carbon Layer and GHG Emissions

**Figure 3:**
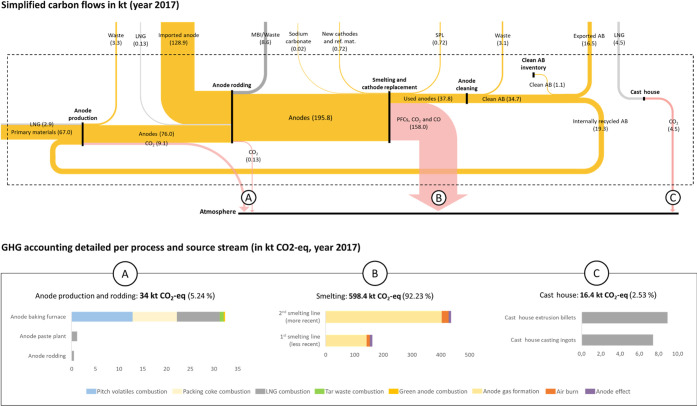
Simplified carbon flows (in kt of carbon) and associated GHG emissions (in kt CO_2_-equiv) in 2017. Fuel flows are shown in light gray, mass balance inconsistency (MBI) in darker gray, GHG emissions flows in red, and other materials in yellow. MBI = mass balance inconsistency; AB = anode butts; PFCs = perfluorinated compounds; LNG = liquid natural gas; ref mat. = refractory materials; SPL = spent pot lining.

Figure 3 presents simplified results of the carbon layer MFA and detailed GHG emissions accounting for the year 2017, obtained by systematically tracking carbon flows within the plant. Results are consistent with the existing literature and previous measurements, such as the emission intensity of the smelting process^44^ and its excess carbon consumption,^42^ CO_2_ to CO ratio in the anode gas from smelting,^45^ and weight loss of the anodes during the baking process.^44^ Detailed Sankey diagrams of the anode plant and anode cleaning subsystems are available in Sections S8 and S9.

### System-Based Indicators

**Figure 4:**
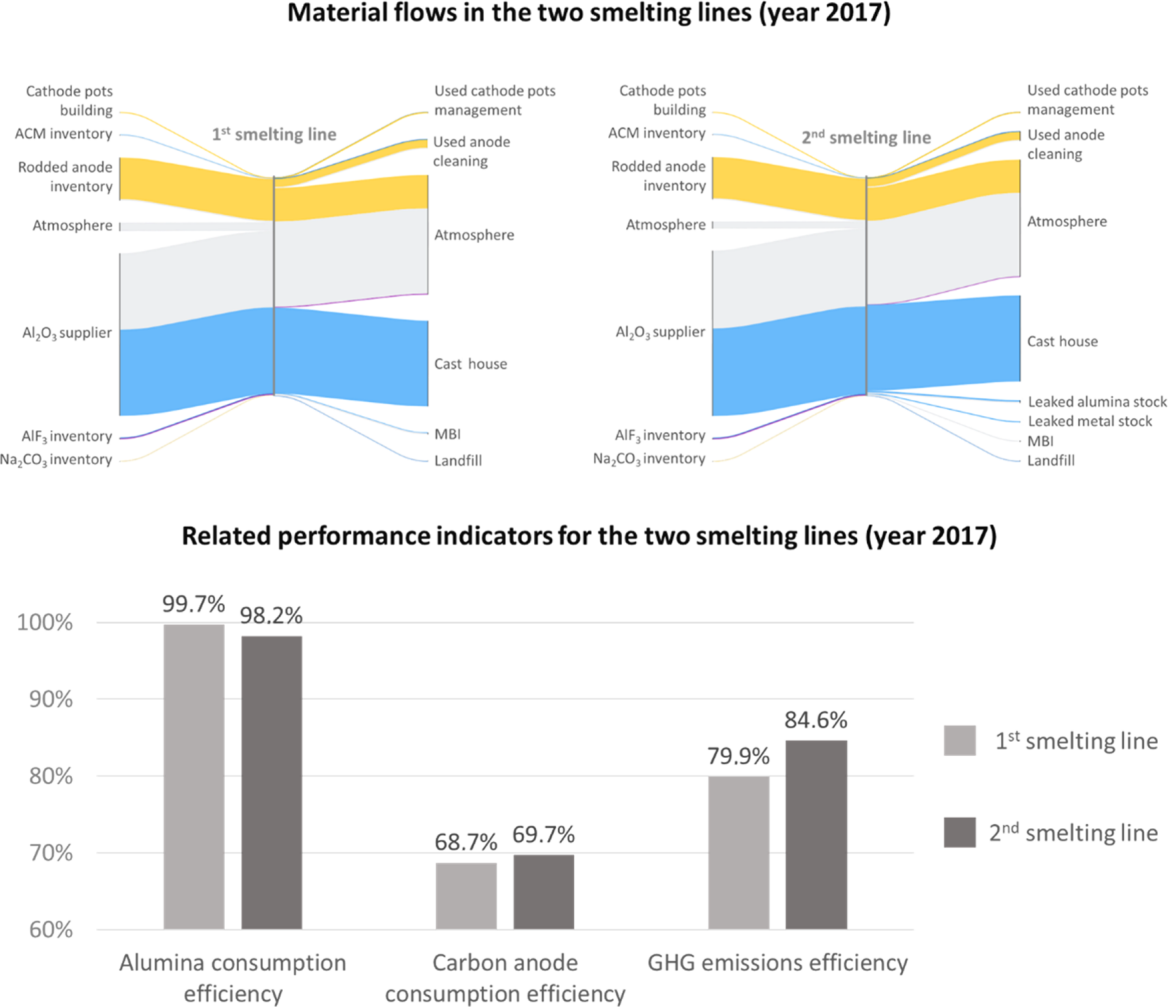
Example of detailed material flow analysis for the smelting processes and the related set of indicators. Upper part: aluminum flows are shown in blue, carbon flows in yellow, fluoride flows in purple, and other materials (mainly oxygen) in light gray. Internal recycling of Al_2_O_3_, AlF_3_, and ACM within the smelting lines is not shown for reasons of simplicity; this choice of resolution does not affect the overall mass balance. A higher resolution might be useful for certain applications, including the development of additional indicators. MBI = mass balance inconsistency.

As illustrated here for the two smelting lines of the plant, our approach enables the design of a set of system-based indicators that integrates both resource efficiency and GHG emission levels. Figure 4 shows that the second smelting line operates closer to the theoretical optimum (see Section S10) when it comes to GHG emissions and carbon consumption. Looking at aluminum extraction from alumina, the first smelting line performs slightly better than the second one. This is due to spillage in the second smelting line, as shown in the Sankey diagram of Figure 4. The real efficiency of the reduction process occurring inside the pots of the second smelting line is hidden by the spill: if it were plugged, all things being equal, it would perform better than the first line.

### Theoretical Emission Reduction Potential of Technological Mitigation Options

**Figure 5:**
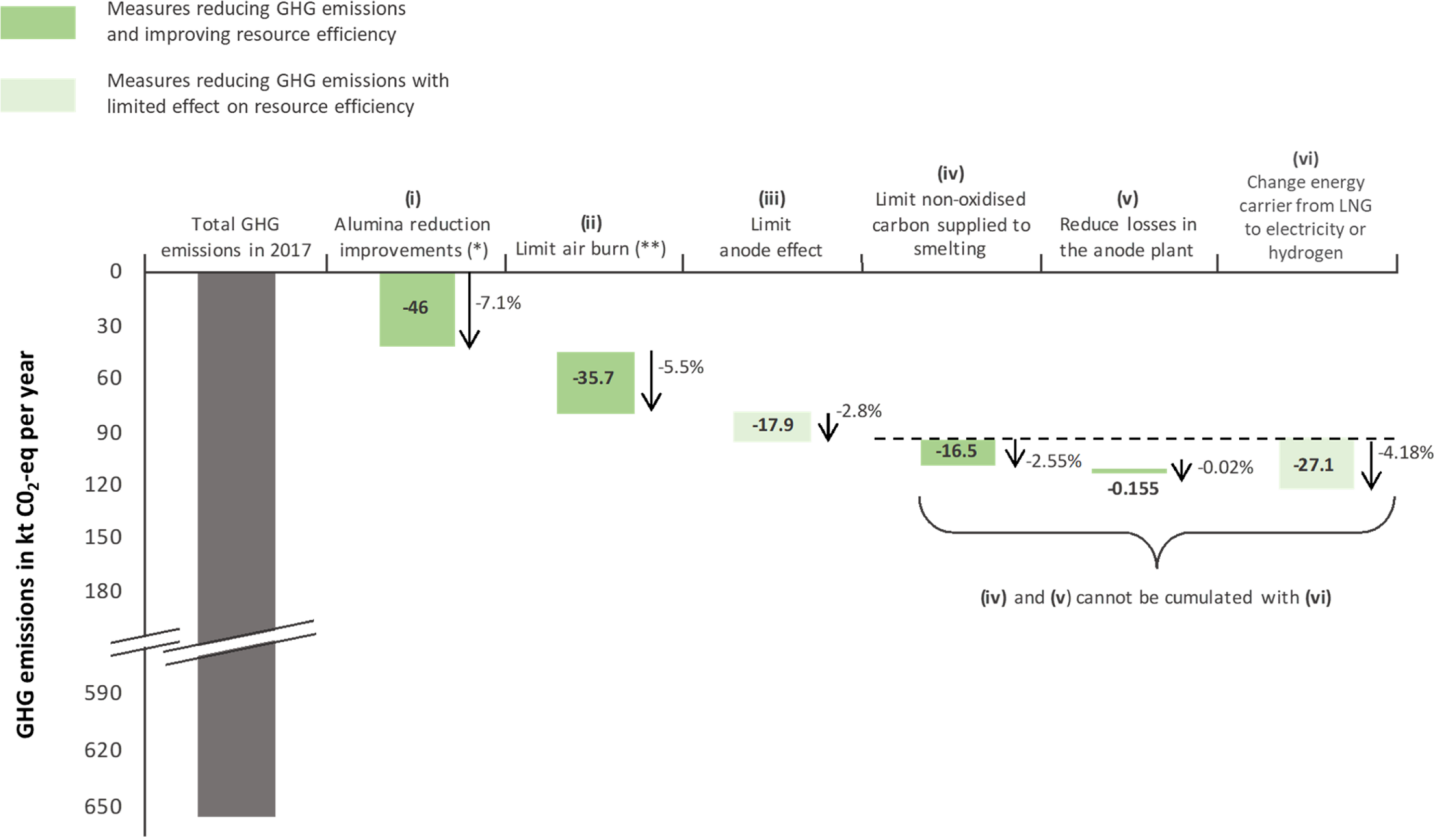
Theoretical yearly GHG emission reduction potential. (*) Alumina reduction improvements refer to increasing the proportion of carbon reacting ideally during smelting and limiting CO_2_ burn and back reaction. (**) Decrease in the amount of carbon oxidized via air burn during smelting and oxidation of carbon monoxide from the exhaust gas with ambient air. LNG = liquid natural gas.

Figure 5 shows the theoretical potential of different options to reduce the yearly GHG emissions of the studied plant. The greatest emission reduction potential lies in improving the smelting process (−116 kt CO_2_-equiv, i.e., 18% decrease compared with 2017 levels).

Improving alumina reduction so that the cells can operate closer to the theoretical reaction had the potential to reduce annual emissions by 7.1% in 2017. Nevertheless, this will be challenging from a technical point of view: the plant studied consumed less than 0.4 kg of carbon per kg of aluminum produced, one of the lowest values reported in the industry.^44,46^ The net carbon consumption could be reduced by increasing the pitch content of the anodes,^47^ given that locally produced anodes contain 13.3% of pitch, while this value ranges from 13 to 18% in the industry.^44^ Additionally, net carbon consumption could be reduced by decreasing the metallic impurity content in the anodes.^47^ This would require improved waste sorting technologies, as we estimated that carbon anode butts recycled in the plant in 2017 contained 1.18% of aluminum impurities after going through the cleaning processes.

Reducing air burn, for example, by covering the upper part of the anodes more carefully to limit contact with oxygen from the ambient air,^40,48,49^ has a great potential to cut direct emissions (−5.5% in 2017). Since it has little influence on the bath chemistry, it might prove easier to implement than improving alumina reduction. Increasing the thickness of the anode cover material (ACM), novel coating techniques, and other technologies could further help to meet this ambition.^48−50^

Industry has focused a lot on reducing AE in the past few decades;^51^ consequently, results showed that reducing it further would have a lower impact than the measures mentioned above to reduce direct GHG emissions. Reducing AE can be achieved by improving computer control of the operating procedures,^52^ but it might be challenging with the current cell technology because the performance of the plant is very close to the industryʼs best practice reported by Cusano et al.^44^ The reduction potential would however be much higher for older or less performant plants.

Waste reduction in the anode plant could cut annual direct emissions by 0.02% (155 kg CO_2_-equiv/year), while changing the energy carrier from LNG to hydrogen or electricity would result in a reduction of 4.18%/year. However, even if the hydrogen option is currently being considered,^53^ the technical feasibility is still uncertain, and benefits would need to be evaluated in a broader system considering electricity/hydrogen production and transport.

### Streams of Waste and Byproducts

**Figure 6:**
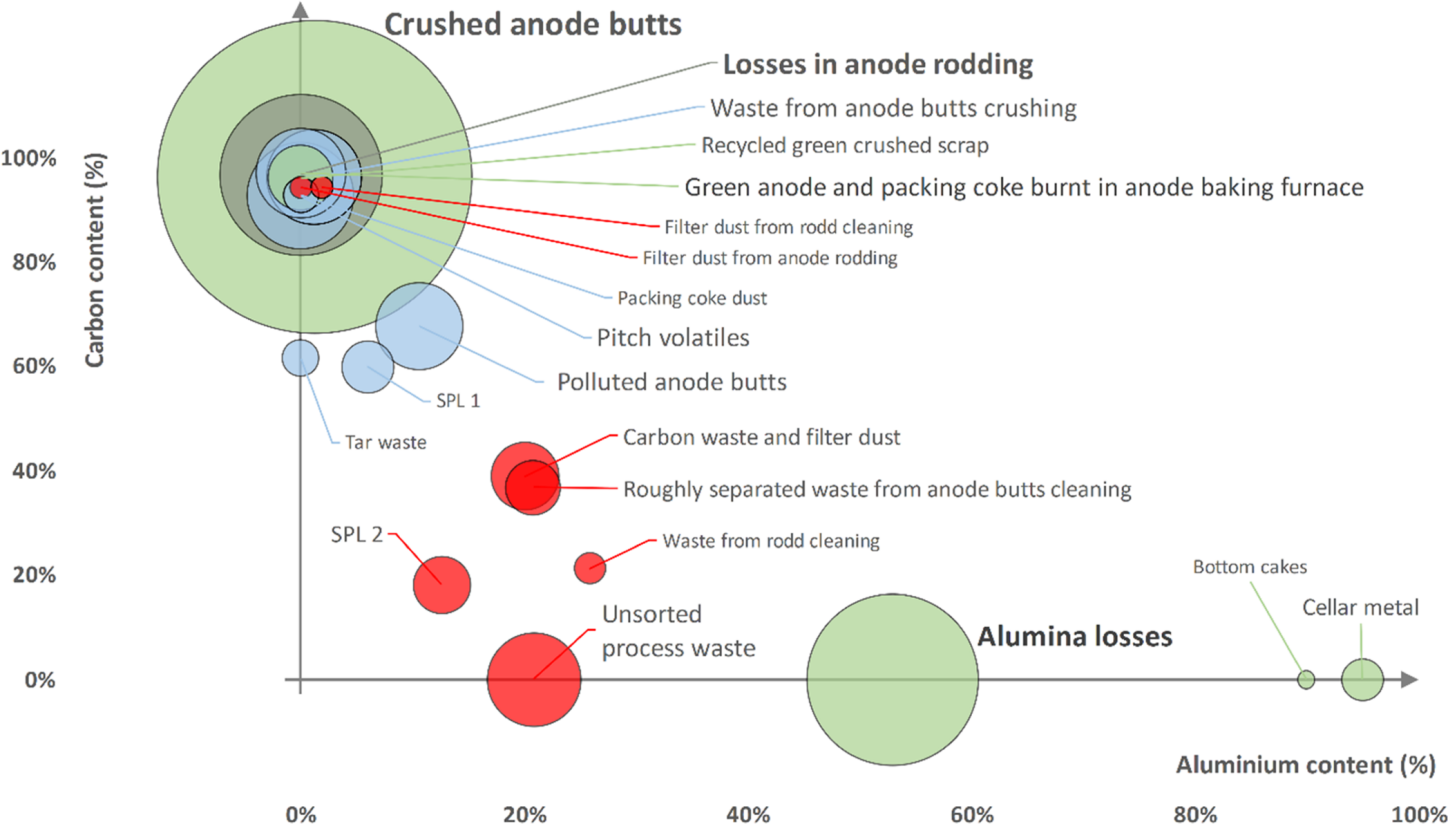
Overview of the different waste flows generated in 2017 according to their Al and C content and their EoL management method. SPL1 = spent potlining first cut, i.e., carbon fraction; SPL2 = spent potlining second cut, i.e., refractory fraction. Color of the dots refers to their EoL management method: green = recycled, blue = energy recovery, red = landfilled. Size of the dots refers to the weight of the waste stream (the bigger dot being 33.5 kt and the smaller 0.4 kt).

Figure 6 shows an overview of the waste streams, their composition (Al and C content), and EoL treatment. We identified clusters of waste/byproducts depending on their composition, which often determines their preferred EoL treatment: (i) the waste containing almost pure carbon is internally recycled, (ii) the waste with a high carbon content (60–70%) and a low aluminum content is used for energy recovery, (iii) the waste containing significant fractions of both carbon and aluminum is landfilled, and (iv) the waste with a high aluminum content and a very low carbon content is externally recycled. This synthesis enables a first crude evaluation of the EoL treatment options for different waste streams. For instance, not all waste flows from the cluster (i) are recycled: although they share the same characteristics in terms of composition, some are used for energy recovery or even landfilled. Similarly, one could investigate to which extent the waste used for energy recovery outside the plant could be used locally as a substitute for imported fuel, thereby decreasing indirect GHG emissions and costs associated with transportation.

## Discussion

### Increase the Robustness and Relevance of GHG Reporting with MFA-Based Accounting

Plant-level MFA enabled the quantification of GHG emissions with a greater level of detail than the EU ETS accounting methodology, shedding light on emissions from each process and breaking down the emissions per source reaction. Compared with the MFA-based GHG accounting, the EU ETS slightly overestimates the total GHG emissions (+4.2%), yet it is difficult to allocate this difference to a specific cause due to the low level of detail provided by the EU ETS accounting. The sensitivity analysis suggests that results are robust for GHG emissions as the most uncertain parameters, including the ratio of CO_2_ to CO in the exhaust gas, have no or very little influence on these flows (Section S7). Hence, neglecting CO emissions from smelting—as done in the EU ETS accounting—seems reasonable to evaluate the total bulk GHG emissions of the plant. Nevertheless, taking CO emissions into account using an MFA-based methodology provides further insights into the causes of the emissions, such as distinguishing between alumina-based anode oxidation and air burn.

Like the EU ETS methodology, our physical accounting approach only considers direct GHG emissions of industrial sites. However, the better understanding of linkages between emissions and material flows is a good starting point for the inclusion of scope 2 and 3 emission inventories, which is needed to avoid problem shifting and design more ambitious strategies. Our study differentiates only two elemental layers, carbon and aluminum, which is sufficient to illustrate the main systemic effects between resource use and GHG emissions. However, additional linkages could be uncovered by considering additional chemical elements, such as fluorine and sodium. Similarly, adding energy and/or exergy layers would allow potential trade-offs between material and energy efficiency to be better quantified.

While the EU ETS methodology only considers aggregate stock changes at the plant level and does not differentiate inventory changes in different parts of the system, MFA-based accounting includes inventories in a more detailed and consistent way. This allows us to explicitly consider the time lag between emissions in different parts of the system and the sales/production, which is better aligned with reality and therefore better suited for tracking performance over time. An illustration is that following the EU ETS methodology, traded quantities of waste are used as a proxy to quantify produced quantities. For instance, inventories of clean anode butts (Figure 3) are often neglected; hence, emission calculation differs from the actual production activity.

MFA-based accounting is also more robust because it enables mass balance consistency checks and facilitates the identification of inconsistencies in different parts of the system. Based on the data available to perform the MFA, the anode rodding process of the plant held a mass balance inconsistency of 8.6 kt of carbon in 2017 (i.e., 8.6 kt of carbon were missing from the outflow of this process). The EU ETS methodology would not enable us to spot this inconsistency and would account for missing outflows from the carbon balance of the plant as emission flows by default—standing for 31.5 kt CO_2_ in the case of the inconsistency mentioned above. On the contrary, further investigations showed that the inconsistency was due to data uncertainty and/or unaccounted solid waste. Ensuring that the material balance of the plant is respected through data reconciliation—made especially possible here by performing a multilayer MFA—reduces the uncertainty of the results. Some of the mass balance inconsistencies may also be attributed to the time resolution chosen. The annual balance applied here is usually sufficient to balance out short-time fluctuations, although some inventories change over longer periods, requiring either a longer balancing period or a higher time resolution for stock accumulation and depletion.

### Reconcile EU ETS and Internal Reporting

The EU ETS methodology is a robust framework to quantify bulk GHG emissions within a reasonable margin of error, while physical accounting provides deeper insights into sources and causes of emissions. The MFA-based tool builds on existing plant-level data to link physical accounting and carbon reporting. Increased granularity and consistency between materials and emission inventories enable us to use the same data to produce the EU ETS reporting and the set of system-based indicators that is used internally to manage performance improvement.

### Identify and Assess Systemic Emission and Resource Efficiency Strategies

Maps of a plantʼs material and energy stocks and flows (metabolism), combined with scenario analysis tools, can help plant managers to identify not only conventional options for direct emissions saving in isolated processes but also systemic solutions considering linkages between emissions, materials, and energy in different processes and at the plant level. For instance, the aluminum industry has historically focused on reducing AE due to direct productivity and environmental benefits, but our results show that there might be a greater potential in the future for reducing air burn and the amount of nonoxidised carbon supplied to the smelters. Additional emission reduction potentials could be identified with extended system boundaries, for example with a better separation of the different material layers in the cast house or by the inclusion of energy flows. While traditional mitigation options have relied on conventional process-oriented areas of research, which tend to focus on processes where emissions and costs are the highest (e.g., electrolysis), the analysis of a plantʼs metabolism can shed light on systemic strategies for emission reduction, an area that is vastly underexplored.

Applying MFA at the plant level also enables the investigation of potentials for improvements beyond the boundaries of the EU ETS GHG accounting, such as reducing indirect GHG emissions and improving resource efficiency via alternative waste and byproducts management (e.g., alumina losses into the basement of the smelter as shown in Figure 6). It highlights issues left out by the EU ETS accounting, which considers all exported carbon-containing waste as carbon stored without introducing any concept of responsibility for the waste producers, making it easier to shift the waste-handling burden downstream in the production line. One could argue that resource efficiency and waste management are out of the scope of the EU ETS accounting, yet research showed that these topics are intrinsically connected with GHG emission mitigation.^54^ For instance, Figure 5 shows that reducing the amount of anode nonoxidized during smelting has the potential to reduce annual emissions in the production phase of the anodes by 16.5 kt CO_2_-equiv, which stands for 2.55% of the direct emissions in 2017.

Physical accounting at the plant level not only unveils potentials to reduce GHG emissions but regards emissions as part of a larger production system. It enables us to investigate the resource efficiency improvements, for instance, via alternative waste and byproducts management, which are not captured by the EU ETS framework. Thereby, it informs long-term strategies for industries to meet the EU ETS targets and reduce yearly emissions.

### Save Costs

GHG accounting is often considered an important cost factor for companies. However, if the accounting tool used has multiple functions and can help identify the most effective options for saving resources and emissions, the accounting tool may also result in cost savings. Hence, the use of plant-level MFA by corporate decision makers might increase the attention put into GHG emission accounting and mitigation by unveiling synergies with resource efficiency improvements. Above, we proposed four (out of six) theoretical emission reduction measures that would also decrease raw material consumption. Our approach also helps industries to meet the emission reduction targets and to lower the costs of emission taxes.

### Conclusion: Implications of Using Physical Accounting in Industrial Sites

While emission reporting and resource efficiency are traditionally analyzed in different systems within a given industrial site, we integrated them in a single framework by studying the plantʼs metabolism and systematically tracking resource and emission flows. We built a tool consistent with the internal reporting system of the company so that once established it can easily be updated and adapted to the needs of plant managers.

Physical accounting based on plant-level MFA has the potential to inform long-term investment strategies for resource efficiency and GHG emission reduction targets to link these strategies with operational management and accounting tools and, in fine, to reach the emission reduction targets set by the EU ETS. If applied widely in industry, this approach opens up the prospect of faster, deeper, and cheaper improvements in resource efficiency and climate change mitigation.

## Supporting Information Available

Methodology for calculation of emission flows; list of assumptions used to quantify the system; EU ETS framework methodologies; theoretical emission reduction potential calculations; list of assumptions used to quantify the system; quantification methods for the different layers and flows of the system; sensitivity analysis methodology and results; detailed Sankey diagrams of the anode plant and anode cleaning subsystems; performance indicator calculations (PDF)

## References

[ref1] Fischedick M.; Roy J.; Abdel-Aziz A.; Acquaye A.; Allwood J. M.; Ceron J.-P.; Geng Y.; Kheshgi H.; Lanza A.; Perczyk D.; Price L.; Santalla E.; Sheinbaum C.; Tanaka K. Industry. In Climate Change 2014: Mitigation of Climate Change, Contribution of Working Group III to the Fifth Assessment Report of the Intergovernmental Panel on Climate Change; Edenhofer O.; Pichs-Madruga R.; Sokona Y.; Farahani E.; Kadner S.; Seyboth K.; Adler A.; Baum I.; Brunner S.; Eickemeier P.; Kriemann B.; Savolainen J.; Schlömer S.; von Stechow C.; Zwickel T.; Minx J. C., Eds.; Cambridge University Press: Cambridge, 2014; p 743.

[ref2] Liu G.; Bangs C. E.; Müller D. B. Stock Dynamics and Emission Pathways of the Global Aluminium Cycle. Nat. Clim. Change 2013, 3, 338–342. 10.1038/nclimate1698.

[ref3] Allwood J. M.; Cullen J. M.; Milford R. L.; McBrien M.; Carruth M. A.; Patel A.; Cooper D.; Moynihan M. Sustainable Materials: With Both Eyes Open; UIT Cambridge Limited, 2012; pp 51–68.

[ref4] Rogelj J.; Shindell D.; Jiang K.; Fifita S.; Forster P.; Ginzburg V.; Handa C.; Kheshgi H.; Kobayashi S.; Kriegler E.; Mundaca L.; Séférian R.; Vilarino M. V. Mitigation Pathways Compatible with 1.5 °C in the Context of Sustainable Development. In Global Warming of 1.5 °C. An IPCC Special Report on the Impacts of Global Warming of 1.5 °C above Pre-industriaL Levels and Related Global Greenhouse Gas Emission Pathways, in the Efforts to Eradicate Poverty; IPCC, 2018.

[ref5] Hill M. R. The European Union’s Emissions Trading Scheme: A Policy Response to the Kyoto Protocol. J. Contemp. Eur. Stud. 2006, 14, 393–410. 10.1080/14782800601102658.

[ref6] European Union: An Emissions Trading Case Study; International Emissions Trading Association, Environmental Defense Fund, CDC Research, 2015.

[ref7] Directive (EU) 2018/410 of the European Parliament and of the Council of 14 March 2018 Amending Directive 2003/87/EC to Enhance Cost-Effective Emission Reductions and Low-Carbon Investments, and Decision (EU) 2015/1814. Off. J. Eur. Union 2018, L76/3–L76/27.

[ref8] Commission Regulation (EU) No 601/2012 of 21 June 2012 on the Monitoring and Reporting of Greenhouse Gas Emissions Pursuant to Directive 2003/87/EC of the European Parliament and of the Council. Off. J. Eur. Union 2012, L181/30.

[ref9] Jacquier G.; Bellassen V. Trendsetter for Companies and Industrial Sites: The EU Emissions Trading Scheme. In Accounting for Carbon: Monitoring, Reporting and Verifying Emissions in the Climate Economy; Bellassen V.; Stephan N., Eds.; Cambridge University Press: Cambridge, 2015; pp 139–189.

[ref10] Tang Q.; Luo L. Carbon Management Systems and Carbon Mitigation. Aust. Accounting Rev. 2014, 24, 84–98. 10.1111/auar.12010.

[ref11] Brander M.; Ascui F. The Attributional-Consequential Distinction and Its Applicability to Corporate Carbon Accounting. In Corporate Carbon and Climate Accounting; Schaltegger S.; Zvezdov D.; Alvarez Etxeberria I.; Csutora M.; Günther E., Eds.; Springer: Cham, 2015; pp 99–120.

[ref12] Liesen A.; Hoepner A. G.; Patten D. M.; Figge F. Does Stakeholder Pressure Influence Corporate GHG Emissions Reporting? Empirical Evidence from Europe. Accounting, Auditing Accountability J. 2015, 28, 1047–1074. 10.1108/AAAJ-12-2013-1547.

[ref13] Andrew J.; Cortese C. Accounting for Climate Change and the Self-Regulation of Carbon Disclosures. Accounting Forum 2011, 35, 130–138. 10.1016/j.accfor.2011.06.006.

[ref14] Heindl P. Transaction Costs and Tradable Permits: Empirical Evidence from the EU Emissions Trading Scheme; ZEW—Leibniz Centre for European Economic Research, 2012.

[ref15] Jaraité J.; Convery F.; Di Maria C. Transaction Costs for Firms in the EU ETS: Lessons from Ireland. Clim. Policy 2010, 10, 190–215. 10.3763/cpol.2009.0659.

[ref16] King K.; Pye S.; Davison S. Assessing the Cost to UK Operators of Compliance with the EU Emission Trading System; Aether Ltd., 2010.

[ref17] Spengler T.; Geldermann J.; Hähre S.; Sieverdingbeck A.; Rentz O. Development of a Multiple Criteria Based Decision Support System for Environmental Assessment of Recycling Measures in the Iron and Steel Making Industry. J. Cleaner Prod. 1998, 6, 37–52. 10.1016/s0959-6526(97)00048-6.

[ref18] Schultmann F.; Engels B.; Rentz O. Flowsheeting-Based Simulation of Recycling Concepts in the Metal Industry. J. Cleaner Prod. 2004, 12, 737–751. 10.1016/S0959-6526(03)00050-7.

[ref19] Porzio G. F.; Fornai B.; Amato A.; Matarese N.; Vannucci M.; Chiappelli L.; Colla V. Reducing the Energy Consumption and CO2 Emissions of Energy Intensive Industries through Decision Support Systems – An Example of Application to the Steel Industry. Appl. Energy 2013, 112, 818–833. 10.1016/j.apenergy.2013.05.005.

[ref20] Belevi H.; Moench H. Factors Determining the Element Behavior in Municipal Solid Waste Incinerators. 1. Field Studies. Environ. Sci. Technol. 2000, 34, 2501–2506. 10.1021/es991078m.

[ref21] Graedel T. E. Material Flow Analysis from Origin to Evolution. Environ. Sci. Technol. 2019, 53, 12188–12196. 10.1021/acs.est.9b03413. 31549816

[ref22] Na H.; Gao C.; Tian M.; Qi Z.; Ye Z. MFA-Based Analysis of CO2 Emissions from Typical Industry in Urban — As a Case of Steel Industry. Ecol. Model. 2017, 365, 45–54. 10.1016/j.ecolmodel.2017.09.023.

[ref23] Xu W.; Cao W.; Zhu T.; Li Y.; Wan B. Material Flow Analysis of CO2 Emissions from Blast Furnace and Basic Oxygen Furnace Steelmaking Systems in China. Steel Res. Int. 2015, 86, 1063–1072. 10.1002/srin.201400228.

[ref24] Wu J.; Wang R.; Pu G.; Qi H. Integrated Assessment of Exergy, Energy and Carbon Dioxide Emissions in an Iron and Steel Industrial Network. Appl. Energy 2016, 183, 430–444. 10.1016/j.apenergy.2016.08.192.

[ref25] Khattak S. H.; Greenough R.; Korolija I.; Brown N. An Exergy Based Approach to Resource Accounting for Factories. J. Cleaner Prod. 2016, 121, 99–108. 10.1016/j.jclepro.2015.12.029.

[ref26] Ball P. D.; Despeisse M.; Evans S.; Greenough R. M.; Hope S. B.; Kerrigan R.; Levers A.; Lunt P.; Oates M. R.; Quincey R.; Shao L.; Waltniel T.; Wheatley C.; Wright A. J. In Modeling Buildings, Facilities and Manufacturing Operations to Reduce Energy Consumption, POMS 23rd Annual Conference, Chicago, 2011.

[ref27] Gonzalez Hernandez A.; Lupton R. C.; Williams C.; Cullen J. M. Control Data, Sankey Diagrams, and Exergy: Assessing the Resource Efficiency of Industrial Plants. Appl. Energy 2018, 218, 232–245. 10.1016/j.apenergy.2018.02.181.

[ref28] Chen W. Q.; Graedel T. E. Anthropogenic Cycles of the Elements: A Critical Review. Environ. Sci. Technol. 2012, 46, 8574–8586. 10.1021/es3010333. 22803614

[ref29] Liu G.; Müller D. B. Mapping the Global Journey of Anthropogenic Aluminium: A Trade-Linked Multilevel Material Flow Analysis. Environ. Sci. Technol. 2013, 11873–11881. 10.1021/es4024404. 24025046

[ref30] Cullen J. M.; Allwood J. M. Mapping the Global Flow of Aluminum: From Liquid Aluminum to End-Use Goods. Environ. Sci. Technol. 2013, 47, 3057–3064. 10.1021/es304256s. 23438734

[ref31] Bertram M.; Ramkumar S.; Rechberger H.; Rombach G.; Bayliss C.; Martchek K. J.; Müller D. B.; Liu G. A Regionally-Linked, Dynamic Material Flow Modelling Tool for Rolled, Extruded and Cast Aluminium Products. Resour., Conserv. Recycl. 2017, 125, 48–69. 10.1016/j.resconrec.2017.05.014.

[ref32] Hansen E. Experience with the Use of Substance Flow Analysis in Denmark. J. Ind. Ecol. 2002, 6, 201–220. 10.1162/108819802766269601.

[ref33] Hatayama H.; Daigo I.; Matsuno Y.; Adachi Y. Assessment of the Recycling Potential of Aluminum in Japan, the United States, Europe and China. Mater. Trans., JIM 2009, 50, 650–656. 10.2320/matertrans.MRA2008337.

[ref34] McMillan C. A.; Moore M. R.; Keoleian G. A.; Bulkley J. W. Quantifying U.S. Aluminum in-Use Stocks and Their Relationship with Economic Output. Ecol. Econ. 2010, 69, 2606–2613. 10.1016/j.ecolecon.2010.08.005.

[ref35] Liu G.; Bangs C. E.; Müller D. B. Unearthing Potentials for Decarbonizing the U.S. Aluminum Cycle. Environ. Sci. Technol. 2011, 45, 9515–9522. 10.1021/es202211w. 21970673

[ref36] Chen W. Q.; Shi L. Analysis of Aluminum Stocks and Flows in Mainland China from 1950 to 2009: Exploring the Dynamics Driving the Rapid Increase in China’s Aluminum Production. Resour., Conserv. Recycl. 2012, 65, 18–28. 10.1016/j.resconrec.2012.05.003.

[ref37] Dai M.; Wang P.; Chen W. Q.; Liu G. Scenario Analysis of China’s Aluminum Cycle Reveals the Coming Scrap Age and the End of Primary Aluminum Boom. J. Cleaner Prod. 2019, 226, 793–804. 10.1016/j.jclepro.2019.04.029.

[ref38] Hannula J.; Llamas J. R. A. G. A. A.; Reuter S. L. M. A. Simulation - Based Exergy and LCA Analysis of Aluminum Recycling: Linking Predictive Physical Separation and Re - Melting Process Models with Specific Alloy Production. J. Sustainable Metall. 2020, 6, 174–189. 10.1007/s40831-020-00267-6.

[ref39] Baccini P.; Brunner P. H. Metabolism of the Anthroposphere; Springer-Verlag: Berlin, 1991; p 157.

[ref40] Aarhaug T. A.; Ratvik A. P. Aluminium Primary Production Off-Gas Composition and Emissions: An Overview. JOM 2019, 71, 2966–2977. 10.1007/s11837-019-03370-6.

[ref41] Kimmerle F. M.; Noel L.; Pisano J. T.; Makay G. I.; Huglen R. COS, CS2 and SO2 Emissions from Prebaked Hall Heroult Cells. Light Metals; TMS, 1997; pp 153–158.

[ref42] Wilkening S. Reflections on the Carbon Consumption of Prebaked Anodes. Essential Readings in Light Metals; John Wiley & Sons, Ltd., 2013; pp 623–632.

[ref43] Solheim A. Inert Anodes—The Blind Alley to Environmental Friendliness?. Light Metals 2018; Springer International Publishing, 2018.

[ref44] Cusano G.; Rodrigo Gonzalo M.; Farrell F.; Remus R.; Roudier S.; Delgado Sancho L. Best Available Techniques (BAT) Reference Document for the Non-Ferrous Metals Industries; EU, 2017.

[ref45] Aarhaug T. A.; Ferber A.; Kjos O.; Gaertner H. Online Monitoring of Aluminium Primary Production Gas Composition by Use of Fourier-Transform Infrared Spectrometry. Light Metals 2014; John Wiley & Sons, Inc., 2014; pp 647–652.

[ref46] International Aluminium Institute. Life Cycle Inventory Data and Environmental Metrics for the Primary Aluminium Industry; International Aluminium Institute, 2017.

[ref47] Khaji K.; Al Qassemi M. The Role of Anode Manufacturing Processes in Net Carbon Consumption. Metals 2016, 6, 128 10.3390/met6060128.

[ref48] Ishak R.; Picard D.; Laroche G.; Ziegler D.; Alamdari H. Application of Boron Oxide as a Protective Surface Treatment to Decrease the Air Reactivity of Carbon Anodes. Metals 2017, 7, 79 10.3390/met7030079.

[ref49] Shakhrai S.; Polyakov P.; Arkhipov G.; Shaidulin E.; Sman’ A. Anode Mass Cover as an Aluminum Electrolyzer Subsystem. Metallurgist 2015, 58, 1128 10.1007/s11015-015-0051-3.

[ref50] Jahedi M.; Oh A.; Gulizia E.; Gulizia S.; Jassim Malallah A.; Al Jallaf M.; Al Jabbri N.; Al Zarouni A. In Anode Coating to Prevent Airburn in Aluminium Smelters, Proceedings of the Light Metals 2009—TMS 2009: 138th Annual Meeting and Exhibition, San Francisco, CA, Feb 15–19, 2009.

[ref51] International Aluminium Institute. Results of the 2017 Anode Effect Survey; International Aluminium Institute, 2018.

[ref52] Bernstein L.; Roy J.; Delhotal K. C.; Harnisch J.; Matsuhashi R.; Price L.; Tanaka K.; Worrell E.; Yamba F.; Fengqi Z. Industry. In Climate Change 2007: Mitigation; Contribution of Working Group III to the Fourth Assessment Report of the Intergovernmental Panel on Climate Change;Metz B.; Davidson O. R.; Bosch P. R.; Dave R.; Meyer L. A., Eds.; Cambridge University Press: Cambridge, 2007.

[ref53] Andersen I. Hydros Aluminiumsverk Er Blant Landets Største Utslippspunkt (Hydro’s Smelters Are among the Biggest Emission Points in the Country) [Online]; Teknisk Ukeblad, 2021 (accessed Dec 11, 2011).

[ref54] Allwood J. M.; Cullen J. M.; Milford R. L.; McBrien M.; Carruth M. A.; Patel A.; Cooper D.; Moynihan M. Sustainable Materials: With Both Eyes Open; UIT Cambridge Limited, 2012; pp 277–284.

