# Supplementary material for: Systemic Approaches for Emission Reduction in Industrial Plants Based on Physical Accounting: Example for an Aluminum Smelter
Source: Environ Sci Technol. 2022 Jan 19;56(3):1973–82. doi: 10.1021/acs.est.1c05681 (PMC8812049; doi:10.1021/acs.est.1c05681)
Supplement: Supplementary file 1 — es1c05681_si_001.pdf [file es1c05681_si_001.pdf]

Supporting Information for:

Systemic approaches for emission reduction in industrial plants based on physical accounting: example for an aluminium smelter

Romain G. Billy[1*], Louis Monnier[12], Even Nybakke[3], Morten Isaksen[3], Daniel B. Müller[1]

[1]Industrial Ecology Programme, Department of Energy and Process Engineering, Norwegian University of Science and Technology (NTNU), Høgskoleringen 5, 7034 Trondheim, Norway

[2]Utopies, 25 Rue Titon, 75011 Paris, France

[3]Hydro Aluminium, Drammensveien 264, 0283 Oslo , Norway

*Corresponding author: romain.billy@ntnu.no

1. Methodology for calculation of emission flows 2

1.1. Calculation of CO2 and CO emissions from anode gases during smelting 2

1.2. Calculation of CO2 and CO emissions from exhaust gases during smelting 3

1.3. Carbon losses from air burn 5

1.4. Emissions during anode production 5

2. EU ETS framework methodologies 6

2.1. Mass balance methodology in the EU ETS accounting framework 6

2.2. Standard methodology in the EU ETS accounting framework 6

2.3. Slope methodology in the EU ETS PFCs accounting framework 6

3. Theoretical emission reduction potential calculations 8

4. List of assumptions used to quantify the system 9

5. Quantification methods for the different layers and flows of the system 13

6. Sensitivity analysis methodology 16

7. Sensitivity analysis results 17

8. Detailed Sankey Diagram of the anode plant subsystem 18

9. Detailed Sankey Diagrams of the anode cleaning subsystem 19

10. Performance indicators calculations..20

References..21

1. Methodology for calculation of emission flows

1.1. Calculation of CO2 and CO emissions from anode gases during smelting

For each smelting line *i*, we assume that:

- The ratio of CO_2 to CO ($r_{CO2/CO}$) in mass unit is assumed to be equal to the one measured by Kimmerle et al.[3] in both smelting lines, i.e. $r_{CO2/CO} = 7.8$ (eq. 3) ;
- All the oxygen contained in the reacting alumina is transformed into CO or CO_2;
- There are no other sources of oxygen in the reaction than oxygen contained in alumina.

Which gives the following equations:

$$\begin{cases} mO_{Al2O3red,i} = m_{CO2,i} \times O\%_{CO2} + m_{CO,i} \times O\%_{CO} \ (eq.1) \\ mC_{reductionAl2O3,i} = m_{CO2,i} \times C\%_{CO2} + m_{CO,i} \times C\%_{CO} + mC_{PFC,i} \ (eq.2) \\ m_{CO,i} \times r_{CO2/CO} = m_{CO2,i} \ (eq.3) \end{cases}$$

By solving the system, we find $m_{CO2,i}$ (CO_2 emissions from smelting line *i*), $m_{CO,i}$ (CO emissions from smelting line *i*) and $mC_{reductionAl2O3,i}$ (carbon consumed to reduce alumina in smelting line i):

$$m_{CO,i} = \frac{mO_{Al2O3red,i}}{O\%_{CO} + r_{CO2/CO} \times O\%_{CO2,i}}$$

$$m_{CO2,i} = r_{CO2/CO} \times \frac{mO_{Al2O3red,i}}{O\%_{CO} + r_{CO2/CO} \times O\%_{CO2,i}}$$

$$mC_{reductionAl2O3,i} = \frac{mO_{Al2O3red,i}}{O\%_{CO} + r_{CO2/CO} \times O\%_{CO2,i}} \times \left[r_{CO2/CO} \times C\%_{CO2} + C\%_{CO}\right] + mC_{PFC,i}$$

With:

- $m_{Al2O3red,i}$ is the oxygen contained in the aluminium oxide reduced in smelting line *i*. It is calculated based on the pure molten aluminium output of smelting line *i*, assuming that all the molten aluminium output comes from reduced alumina. With $m_{Al,i}$ as the pure aluminium output from smelting line *i*, we get: $mO_{Al2O3red,i} = \frac{m_{Al,i}}{Al\%_{Al2O3,i})} \times O\%_{Al2O3,i}$
- $Al\%_{Al2O3,i}$ is the aluminium content of alumina.
- $O\%_{Al2O3,i}$is the oxygen content of alumina.
- $O\%_{CO2}$ is the oxygen content of carbon dioxide.

- $O\%_{CO}$ is the oxygen content of carbon monoxide.
- $C\%_{CO2}$ is the carbon content of carbon dioxide.
- $C\%_{CO}$ is the carbon content of carbon monoxide.
- $mC_{PFC,i}$ is the amount of carbon contained in PFCs emissions (calculated according to appendix 4.3).

1.2. Calculation of CO2 and CO emissions from exhaust gases during smelting

We can calculate the composition of the exhaust gas from each smelting line. For each smelting line i, we assume that:

- All the oxygen contained in the reacting alumina and the dioxygen from the ambient air is transformed into carbon monoxide or carbon dioxide (eq. 1);
- All the oxidised carbon (i.e. carbon that is not emitted as PFCs nor present in solid waste) is emitted as carbon monoxide or carbon dioxide (eq. 2);
- The ratio of CO_2 to CO ($r_{CO2/CO}$) in mass unit is assumed to be equal to the one measured by Kimmerle et al.[3] in both smelting lines, i.e. $r_{CO2/CO} = 13.12$ (eq. 3).

Which gives the three following equations:

$$\begin{cases} mO_{Al2O3red,i} + m_{O2,i} = m_{CO2,i} \times O\%_{CO2} + m_{CO,i} \times O\%_{CO} \ (eq. 1) \\ mC_{Anodes,i} - mC_{Waste\ Anodes,i} - mC_{PFC,i} = m_{CO2,i} \times C\%_{CO2} + m_{CO,i} \times C\%_{CO} \ (eq. 2) \\ m_{CO2,i} \times r_{CO2/CO} = m_{CO,i} \ (eq. 3) \end{cases}$$

By solving the system, we find $m_{O2,i}$ (O_2 inflow to smelting line *i*) $m_{CO2,i}$ (CO_2 emissions from smelting line *i*) and $m_{CO,i}$ (CO emissions from smelting line *i*):

$$m_{CO,i} = \frac{mC_{Anodes,i} - mC_{Waste\ Anodes,i} - mC_{PFC,i}}{C\%_{CO} + r_{CO2/CO} \times C\%_{CO2,i}}$$

$$m_{CO2,i} = r_{CO2/CO} \times \frac{mC_{Anodes,i} - mC_{Waste\ Anodes,i} - mC_{PFC,i}}{C\%_{CO} + r_{CO2/CO} \times C\%_{CO2,i}}$$

$$m_{O2,i} = \left[\frac{mC_{Anodes,i} - mC_{Waste\ Anodes,i} - mC_{PFC,i}}{C\%_{CO} + r_{CO2/CO} \times C\%_{CO2,i}}\right] \times \left(O\%_{CO} + r_{CO2/CO} \times O\%_{CO2,i}\right) - mO_{Al2O3red,i}$$

With:

- $m_{Al,i}$ is the pure aluminium output from smelting line *i*.
- $m_{Al2O3red,i}$ is the oxygen contained in the aluminium oxide reduced in smelting line *i*. It is calculated based on the pure molten aluminium output of smelting line *i*, assuming that all the molten aluminium output comes from reduced alumina. With $m_{Al,i}$ as the pure aluminium output from smelting line *i*, we get: $mO_{Al2O3red,i} = (m_{Al,i}/Al\%_{Al2O3,i}) \times O\%_{Al2O3,i}$
- $Al\%_{Al2O3,i}$ is the aluminium content of alumina.
- $O\%_{Al2O3,i}$is the oxygen content of alumina.
- $mC_{Anodes,i}$ is the carbon contained in the anodes supplied to smelting line *i*.
- $mC_{Waste\ Anodes,i}$ is the carbon contained in the used anodes from smelting line *i*.
- $O\%_{CO2}$ is the oxygen content of carbon dioxide.

- $O\%_{CO}$ is the oxygen content of carbon monoxide.
- $C\%_{CO2}$ is the carbon content of carbon dioxide.
- $C\%_{CO}$ is the carbon content of carbon monoxide.
- $mC_{PFC,i}$ is the amount of carbon contained in PFCs emissions (calculated according to appendix 4.3).

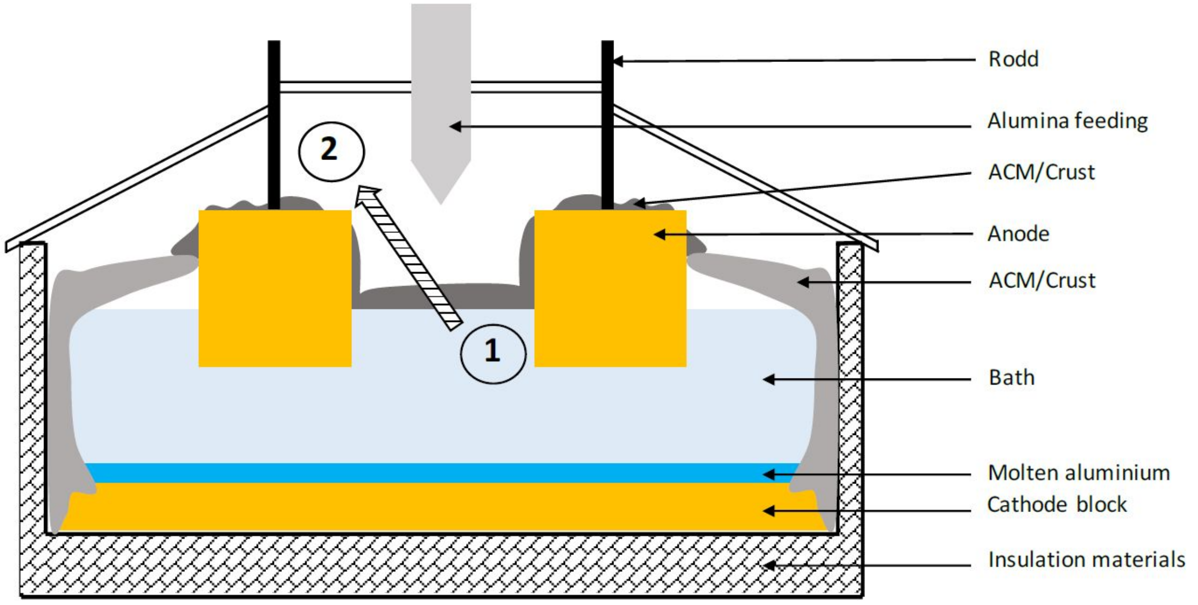

1 **Anode gas formation :** $m_{CO2/CO}$= 7.88

$$\begin{cases} Al_2O_3 + 1.5C = 2Al + CO_2 \text{ (eq. 1a)} \\ 2Al + 3CO_2 = 3CO + Al_2O_3 \ (eq. 1b) \\ C + CO_2 = 2CO \ (eq.1c) \end{cases}$$

2 **Exhaust gas formation :** $m_{CO2/CO}$= 13

$$\begin{cases} 2C + O_2 = 2CO \ (eq. 2a) \\ C + O_2 = CO_2 (eq.2b) \\ 2CO + O_2 = 2CO_2 (eq.2c) \end{cases}$$

Figure S1: *Schematic representation of a pot and main reactions that lead to exhaust gas formation. PFCs formation is not shown but would occur in step 1 (anode gas formation). Ratios of CO_2 to CO are expressed in tons/tons as measured by Kimmerle et al.*[3]

1.3. Carbon losses from air burn

Additionally, because we know the amount of carbon in the anode gas (i.e. oxidised to reduce alumina or in PFCs), and the total amount of carbon emitted to the atmosphere in a given smelting line, we can infer the amount of carbon lost to air burn:

$$mC_{airburn,i} = mC_{Total\ emitted} - mC_{redAl2O3,i} - mC_{PFC,i}$$

Where:

- $mC_{redAl2O3,i}$ is the amount of carbon consumed to reduce alumina in smelting line *i* (calculated according to method shown supra);
- $mC_{PFC,i}$ is the amount of carbon contained in PFCs emissions (calculated according to appendix 3);
- $mC_{Total\ emitted}$ is the total amount of carbon emitted to the atmosphere in a given smelting line, calculated via mass balance of the carbon layer:

$$mC_{Total\ emitted} = \sum C\ inputs_i - \sum C\ in\ solid\ waste_i$$

1.4. Emissions during anode production

Results from the MFA enable to quantify the amount of GHG emissions occurring in each process of the anode plant (anode paste plant, anode baking furnace and anode rodding station) per ton of carbon output.

Table S1: Direct GHG emissions during anode production

Values in kg CO_2-eq per ton of carbon output from each process

	GHG emissions per unit output
Anode paste plant	14.6
Anode baking furnace	425
Anode rodding station	2.4

Summing all the data in Table S1 indicates that the production of one ton of pure carbon (contained in an anode) emits 441 kg CO_2-eq. Hence, assuming that the imported anodes are produced with the same carbon-intensity as the locally produced anodes, one can calculate that the production of 37.8 kt of carbon contained in non-oxidised anodes emits $37.8 \times 0.441 = 16.5$ kt CO_2-eq. In reality, imported anodes are likely to have a greater carbon intensity during their production phase than the ones produced in the plant studied given that their performances are at the industry's best practice.[5] This could be due to the relatively low carbon-intensity of the fuel used by the plant compared with other production sites in the world. Hence this number might be underestimated.

2. *EU ETS framework methodologies*

2.1. Mass balance methodology in the EU ETS accounting framework

This methodology is applied to calculate the emissions associated with anode production (pitch, coke) and anode consumption in the electrolysis. Moreover, when a material is leaving the boundaries of the mass balance (i.e. exported waste, etc.) then its emissions are calculated and accounted for as a negative emission. The carbon dioxide emissions associated with a given source stream *i* are:

$$E_i(tCO_2) = Activity_i \times Concentration_i \times\ 3{,}664$$

Where:

- $Activity_i$: is the the activity data associated with the source stream *i*, i.e. it is the amount of material entering or leaving the boundaries of the mass balance (in tons).
- $Concentration_i$: is the carbon content of the source stream *i* (in %).
- 3,664 : is a conversion factor from tons of carbon to tons of CO_2 (in tCO_2/tC).

Source: European Commission (2012, Article 25)[4]

2.2. Standard methodology in the EU ETS accounting framework

This methodology is used to calculate the emissions associated with fuel combustion.

$$E_i(tCO_2) = Activity_i \times Emission\ factor_i \times\ Oxidation\ factor_i$$

Where:

- $Activity_i$: is the activity data associated with the source stream *i*, i.e. it is the amount of fuel combusted expressed as gigajoules based on net calorific value.
- $Emission\ factor_i$: is the emission factor of the source stream i (in tCO_2/TJ).
- $Oxidation\ factor_i$: is the oxidation factor associated with combustion of fuel i (in %)

Source: European Commission (2012, Article 24)[4]

2.3. Slope methodology in the EU ETS PFCs accounting framework

The slope methodology calculates the PFCs emissions (CF4 and C2F6) occurring during anode effect at a primary aluminium smelter as follows:

$$PFC_{tot}^{em} = \frac{PFC_{duct}^{em}}{Collection\ efficiency}$$

$$PFC_{duct}^{em} = \ CF_4^{em} \times\ GWP_{CF4} + C_2F_6^{em} \times\ GWP_{C2F6}$$

$$CF_4^{em} = AEM \times \frac{SEF_{CF4}}{1000} \times Pr_{Al}$$

$$C_2F_6^{em} = CF_4^{em} \times F_{C2F6}$$

Where:

- PFC_{tot}^{em} is the total calculated emissions of PFCs expressed in CO_2 equivalent.
- PFC_{duct}^{em} is the emissions of PFCs calculated at the duct or stack expressed in CO_2 equivalent.
- $Collection\ efficiency$ is the collection efficiency of the duct or stack (ratio between the emissions occurring at the duct or stack and the fugitive emissions, in %). Collection efficiency is set to 1 in this study.
- GWP_{CF4} , GWP_{C2F6} are the global warming potentials of CF_4 and C_2F_6, respectively 6,500 t CO2(e)/t CF_4 and 9,200 t CO_2(e)/t C_2F_6 (European Commission, 2012, Annex VI Table 6)
- CF_4^{em}, $C_2F_6^{em}$ are the calculated emissions of CF_4 and C_2F_6 at the duct or stack, expressed in tons of CF_4 and C_2F_6 respectively.
- AEM are the Anode effect minutes/cell-day, i.e. the frequency of anode effects multiplied by the average duration of anode effects.
- SEF_{CF4} is the slope emission factor. One can use the technology-specific emission factor provided by the European Commission or determine installation-specific emissions factor via measurements.
- Pr_{Al} is the annual production of primary aluminium (in tons)
- F_{C2F6} is the emission factor of C_2F_6, i.e. the amount of C_2F_6 emitted with regards to the amount of CF_4 emitted (in tC_2F_6 / tCF_4). One can use the technology-specific emission factor provided by the European Commission or determine installation-specific emissions factor via measurements.

Subsequently we get for each smelting line i:

$$m_{PFC,i} = CF_4^{em,i} + C_2F_6^{em,i}$$
$$mC_{PFC,i} = CF_4^{em,i} \times C\%_{CF4} + C_2F_6^{em,i} \times C\%_{C2F6}$$

- $mC_{PFC,i}$ is the amount of carbon contained in PFCs emissions.
- $C\%_{CF4}$ is the carbon content of CF_4.
- $C\%_{C2F6}$ is the carbon content of C_2F_6.

Source: European Commission (2012, Annex IV, Section 8)[4].

3. Theoretical emission reduction potential calculations

The emission reduction potentials were calculated as presented infra.

(i) Alumina reduction improvements:

The theoretical emission reduction potential is equal to the difference between the GHG emissions in the anode gas (calculated according to appendix 5.1) and the theoretical GHG emissions from alumina reduction.

(ii) Limit air burn and oxidation of carbon monoxide from the exhaust gas with the ambient air:

The theoretical emission reduction potential is equal to the GHG emissions in the exhaust gas (calculated according to appendix 5.2) minus the GHG emissions in the anode gas (calculated according to appendix 5.1).

(iii) Limit anode effect:

The theoretical emission reduction potential is equal to the GHG emissions due to AE in both smelting line (calculated according to appendix 4.3).

(iv) Limit non-oxidized carbon supplied to smelting:

The theoretical emission reduction potential is equal to the amount of carbon non-oxidized during smelting multiplied by the GHG emissions due to anode production in kg CO_2-eq per kg of C output, calculated according to appendix 5.4.

(v) Reduce losses in the anode plant:

The theoretical emission reduction potential is calculated according to table S13 in appendix 7.

(vi) Change energy carrier from LNG to electricity:

The theoretical emission reduction potential is equal to the GHG emissions due to combustion of LNG in 2017, quantified according to the MFA methodology (see Table S3 assumption A1 and Table S4 assumption A2).

4. List of assumptions used to quantify the system

The following tables S3 and S4 explain the assumptions made to quantify the system. The level of

uncertainty associated with each assumption has been evaluated as in Table S2. Due to the overall

high data availability and quality and low level of uncertainty, the mass balance calculation method was assigned a medium-low uncertainty level.

Table S2: Classification of the level of uncertainty of the assumptions

Level of uncertainty			
**Low**	**Medium-low**	**Medium**	**High**
Primary data and assumptions with very low level of uncertainty	Mass balance	Primary data and assumptions with medium level of uncertainty	Assumptions with high level of uncertainty

Table S3: Assumptions related with carbon and aluminium content of goods

Name	Assumption	Level of uncertainty
A1	LNG is assumed to be pure methane (CH_4): $C\%_{LNG} = \frac{M_C}{M_C + 4 \times M_H}$	Low
A3	Anode scrap flow from the anode paste plant is assumed to have a carbon content equal to the weighted average of the primary carbon materials input of the process (crushed butts, pitch and petroleum coke).	Low
A4	Locally produced baked anode are assumed to have the same carbon content as locally produced green anodes. Likewise, locally produced rodded anodes are assumed to have the same carbon content as locally produced baked anodes.	Medium
A6	Total rodded anodes (RA) flow is assumed to have a carbon content equal to the weighted average of the one of the imported (imp) and locally produced (lp) rodded anodes: $C\%_{total\ RA\ flow} = \frac{C\%_{lp} \times Locally\ produced\ RA + C\%_{imp} \times Imported\ RA}{Locally\ produced\ RA + Imported\ RA}$	Low
A7	Carbon content of the rodded anodes leaving the inventory (i.e. being used for electrolysis) is the same as the average carbon content of the rodded anodes in the inventory. Aluminium content is assumed to be 0%.	Low
A8	Composition of Anode Cover Material (ACM) is assumed to be 50% bath material and 50% aluminium oxide based on Hydro's documents and existing literature [35]. This gives a carbon content of 0% and an aluminium content of 33%: $ACM = \begin{cases} 50\%\ cryolite\ Na_3AlF_6 \\ 50\%\ aluminium\ oxide\ Al_2O_3 \end{cases}$ $ACM = \begin{cases} C\%_{ACM} = 0 \\ Al\%_{ACM} = 0.5 \times Al\%_{Na3AlF6} + 0.5 \times Al\%_{Al2O3} \end{cases}$	Low
A9	Based on Mikša, et al. [1] and von Krüger [2], carbon content of SPL1 and SPL2 is assumed to be respectively 60% and 18.2%, aluminium content of SPL1 and SPL2 is assumed to be respectively 6% and 12.6%.	Medium
A10	Hydro's EU ETS reporting gives an average value of carbon content for tar waste in anode paste plant and anode baking furnace processes. This average value was used to quantify the tar waste in both processes without trying to further detail the distribution of the carbon in the tar waste amongst the two processes.	Medium
A11	Pitch volatiles burnt in the anode baking furnace are assumed to have the same carbon content as the pitch consumed in the plant to make anode paste.	Medium

Name	Assumption	Level of uncertainty
A15	Carbon content of anode butts (AB) internally recycled is assumed to be the average carbon content of the exported clean butts from the EU ETS reporting: $$C\%_{internally\ recycled\ AB} = \sum_{AB\ importers\ (i)} (C\%_i \times AB\ exported_i) / \sum_{AB\ importers\ (i)} (AB\ exported_i)$$	Low
A17	Sodium carbonate, aluminium oxide and aluminium fluoride used in electrolysis are assumed to be pure Na_2CO_3, Al_2O_3 and AlF_3 respectively: $$Sodium\ carbonate: \begin{cases} C\%_{Na2CO3} = \frac{M_C}{2 \times M_{Na} + M_C + 3 \times M_O} \\ Al\%_{Na2CO3} = 0 \end{cases}$$ $$Aluminium\ oxide: \begin{cases} C\%_{Al2O3} = 0 \\ Al\%_{Al2O3} = \frac{2 \times M_{Al}}{2 \times M_{Al} + 3 \times M_O} \end{cases}$$ $$Aluminium\ fluoride: \begin{cases} C\%_{AlF3} = 0 \\ Al\%_{AlF3} = \frac{M_{Al}}{M_{Al} + 3 \times M_F} \end{cases}$$	Low
A19	Based on Hydro's documents, aluminium content of spilled metal in smelting line 1 and bottom cakes are assumed equal to 95% and 90% respectively, their carbon content is set to 0%.	Low
A20	Unsorted process waste from smelting is assumed to be constituted of 20% pure aluminium oxide and 80% bath material (which gives an aluminium content of around 21% and a carbon content of 0%): $$Unsorted\ process\ waste\ from\ smelting = \begin{cases} 80\%\ cryolite\ Na_3AlF_6 \\ 20\%\ aluminium\ oxide\ Al_2O_3 \end{cases}$$ $$Unsorted\ process\ waste\ from\ smelting = \begin{cases} C\%_{ACM} = 0 \\ Al\%_{ACM} = 0.8 \times Al\%_{Na3AlF6} + 0.2 \times Al\%_{Al2O3} \end{cases}$$	High
A21	Molten aluminium outflow from the electrolysis is assumed to be pure aluminium.	Low
A23	Everything that is not pure carbon in the used anodes is assumed to be pure ACM.	Low
A24	Refractory material inflow for cathode pots is assumed to be the same as SPL2 produced during the year (for total weight, carbon and aluminium content).	High
A26	When necessary, carbon content of the stock of a given process was assumed equal to the weighted average carbon content of the inflow to this process: $$C\%_{materials\ in\ stock\ k} = \sum_{Inflows\ (i) to\ k} (C\%_i \times Materials\ to\ k_i) / \sum_{Inflows\ (i) to\ k} (Materials\ to\ k_i)$$	Low

Table S4: Miscellaneous assumptions

Name	Assumption	Level of uncertainty
A2	LNG combustion is assumed to be complete and to produce only carbon dioxide, i.e. all the carbon in the burnt LNG is emitted back to the air in the form of CO_2.	Low
A5	Only one number is given in the EU ETS reporting for filter dust from both anode rodding process and rod cleaning process. Based on interviews with Hydro's staff, it was assumed that half of the filter dust produced was from the anode rodding process, the other half being from the rod cleaning process.	High
A12	Spent Potlining first and second cut (SPL1 and SPL2) quantities were calculated based on the following estimates made by Hydro's staff:<br>$Parameters\ smelting\ line\ L1: \begin{cases} Number\ of\ cells\ shut\ down\ in\ 2017 = 16\ cells \\ SPL1\ =\ 30\ tons/ce \\ SPL2\ =\ 36\ tons/cell \end{cases}$<br>$Parameters\ smelting\ line\ L2: \begin{cases} Number\ of\ cells\ shut\ down\ in\ 2017 = 20\ cells \\ SPL1\ =\ 20\ tons/ce \\ SPL2\ =\ 24\ tons/cell \end{cases}$<br>$SPL1\ produced\ in\ 2017: \begin{cases} 16 \times 30\ = 480\ tons\ in\ L1 \\ 20 \times 20\ = 400\ tons\ in\ L2 \end{cases}$<br>$SPL2\ produced\ in\ 2017: \begin{cases} 16 \times 36\ = 576\ tons\ in\ L1 \\ 20 \times 24\ = 480\ tons\ in\ L2 \end{cases}$	High
A13	Graphite cathodes are assumed to have a carbon content of 100% and the total amount of carbon contained in the cathodes are assumed to be equal to the quantity of carbon in the SPL1 produced.<br>$Cathode_{Carbon\ layer} = SPL1_{Carbon\ layer} = Cathode_{Goods\ layer}$	High
A14	Total waste of used anodes is spitted between the two smelting lines using molten aluminium output ratios from the two lines:<br>$Split\ between\ the\ two\ smelting\ lines: \begin{cases} k_{L1} = \dfrac{Al\ output_{L1}}{Al\ output_{L1}\ + Al\ output_{L2}} \\ k_{L2} = 1 - k_{L1} \end{cases}$<br>$Waste\ anodes\ from\ i\ =\ k_i \times\ Total\ waste\ anodes,\ i\ \in \{L1, L2\}$	Medium
A18	Exhaust gases from the electrolysis are assumed to be PFCs emissions (calculated with the EU ETS slope methodology presented in appendix 4.3), CO_2 and CO. This assumption is consistent with measured values in similar smelting lines[3] where other gases have proven to be present in negligible fraction.<br>Full methodology for calculation of CO_2 and CO emissions is presented in appendix 5.2 and is based on the assumption that the ratio of CO_2 to CO emitted is the same as the one measured by Kimmerle et al.[3] in a similar pot as the one used by Hydro in Sunndal, i.e. $\frac{CO2}{CO} = 13.12$ (tons of CO_2 / tons of CO) in the exhaust gas.<br>Part of the carbon anode reacts with O_2 from the ambient air. O_2 inflow to the smelting lines is quantified as shown in appendix 5.2.	Low
A22	Total amount of bottom cakes produced is split between the two lines using spent potlining output ratios from the two lines.<br>$Split\ betweent\ the\ two\ smelting\ lines: \begin{cases} q_{L1} = \dfrac{SPL\ output_{L1}}{SPL\ output_{L1}\ + SPL\ output_{L2}} \\ q_{L2} = 1 - q_{L1} \end{cases}$<br>$Bottom\ cakes\ from\ i\ =\ q_i \times\ Total\ bottom\ cakes,\ i\ \in \{L1, L2\}$	High

Name	Assumption	Level of uncertainty
A25	Total new pots flow is assumed equal to the sum of new refractory material flow and cathode flow. The new pots flow and used cathode flow are split between the two lines using the fraction of SPL waste generated in each line.<br>$Total\ new\ pots\ =\ Refractory\ materials\ +\ New\ cathodes$<br>$Split\ betweent\ the\ two\ smelting\ lines: \begin{cases} q_{L1} = \frac{SPL\ output_{L1}}{SPL\ output_{L1}\ + SPL\ output_{L2}} \\ q_{L2} = 1 - q_{L2} \end{cases}$<br>$New\ pots\ to\ i\ =\ q_i \times\ Total\ new\ pots,\ i\ \in\ \{L1, L2\}$<br>$Used\ pots\ from\ i\ =\ q_i \times\ Total\ used\ pots,\ i\ \in\ \{L1, L2\}$	High
A26	ACM inflows to both smelting lines is assumed to be equal to the amount of ACM delivered by both lines (i.e. total amount of ACM delivered split between the two smelting lines with assumption A14).	Medium

5. *Quantification methods for the different layers and flows of the system*

The tables infra summarize how each flow has been quantified for the relevant layer in the different parts of the system (anode production, smelting lines, casthouse, used anode cleaning and cathode replacement). A colour code referring to the classification presented in Table S2 enables to grasp the level of uncertainty associated with each assumption used to quantify the flows.

Table S5: Quantification method of the anode production flows

Flows	Layer		
	Goods	Aluminium	Carbon
Imported pitch and coke, inflows of carbon materials to the anode paste plant (pitch, coke, crushed recycled butts) and the anode baking furnace (packing coke).	Primary data	–	Primary data
Liquid Natural Gas (LNG) inflow to anode paste plant, anode baking furnace and anode rodding.	Primary data	–	Assumption n°1
Tar waste from the anode paste plant and anode baking furnace	Primary data	–	Assumption n°10
Exhaust gas from the anode baking furnace	N/A	–	Mass balance
Exhaust gas from the anode paste plant, exhaust gas from the anode rodding process (all due to LNG combustion)	Assumption n°2	–	Assumption n°2
Pitch volatiles burnt in the anode baking furnace	Primary data	–	Assumption n°11
Recycled green crushed scrap in the anode paste plant	Primary data	–	Assumption n°3
Flow of butts entering the butts crushing process	Mass balance	Assumption n°8, assumption n°23	Mass balance
Changes in crushed butts dust inventory and exported filter dust generated in butts crushing process	Primary data	Assumption n°8, assumption n°23	Primary data and assumption n°26
Filter dust produced from butts crushing process	Mass balance	Assumption n°8, assumption n°23	Mass balance
Flow of green anode leaving the anode paste plant, flow of green anode entering the anode baking furnace	Primary data	–	Mass balance (carbon content found with mass balance of anode paste plant process)
Filter dust from anode rodding process	Primary data and assumption n°5	–	Primary data and assumption n°5
Carbon material losses in anode rodding process	N/A	–	Mass balance
Baked anode flow from anode baking furnace to inventory, baked anode flow to rodded anode inventory	Primary data	–	Assumption n°4
Packing coke dust from the anode baking furnace	Primary data	–	Primary data
Rodded anodes flow to inventory	Primary data	–	Assumption n°6
Imported baked anodes flow to inventory and to anode rodding process	Primary data	–	Primary data

Table S6: Quantification method of the smelting flows

Flows	Layers		
	Goods	Aluminium	Carbon
Rodded anodes inflow to the smelting lines	Primary data	Assumption n°7	Assumption n°6, assumption n°7
Aluminium fluoride and sodium carbonate inflow to the electrolysis lines, imported sodium carbonate, aluminium oxide inflow to the smelting lines	Primary data	Assumption n°17	Assumption n°17
Molten aluminium outflow from the smelting lines	Primary data	Assumption n°21	Assumption n°21
Tap out metal from SU4	Primary data	Assumption n°19	Assumption n°19
Unsorted process waste from the smelting lines	Primary data	Assumption n°20	Assumption n°20
Exhaust gas from the smelting lines	Assumption n°18 and results from the carbon layer	Assumption n°18	Mass balance
Oxygen inflow from the ambient air to the smelting lines	Assumption n°18 and results from the carbon layer	–	–
Used pots flows from the smelting lines	Assumption n°25	Assumption n°25	Assumption n°25
New pots flows to the smelting lines	Assumption n°25	Assumption n°25	Assumption n°25
Used anodes flows from the smelting lines	Assumption n°14 and mass balance	Assumption n°8, assumption n°23	Assumption n°14 and mass balance
ACM inflows to the smelting lines	Assumption n°8, assumption n°26	Assumption n°8, assumption n°26	Assumption n°8, assumption n°26
Alumina losses to SU4's cellar	Mass balance (Aluminium layer) and assumption n°A17	Mass balance (Aluminium layer)	Mass balance (Aluminium layer) and assumption n°A17

Table S7: Quantification method of the casthouse flows

Flows	Layer		
	Goods	Aluminium	Carbon
LNG inflow to metal blending processes	Primary data	–	Assumption n°1
Exhaust gas from metal blending process (due to LNG combustion)	Assumption n°2	–	Assumption n°2

Table S8: Quantification method of the pots replacement flows

Flows	Layers		
	Goods	Aluminium	Carbon
Spent Potlining first and second cut produced during the year (outflow from pots delining, maintenance and relining process)	Assumption n°12	Assumption n°9	Assumption n°9
Spent Potlining first and second cut exported during the year (outflow from inventories)	Primary data	Assumption n°9	Assumption n°9
New cathode flow	Assumption n°13	Assumption n°13	Assumption n°13
New refractory material flow	Assumption n°24	Assumption n°24	Assumption n°24
Bottom cakes flow	Primary data	Assumption n°19	Assumption n°19

Table S9: Quantification method of the used anodes cleaning flows

Flows	Layers		
	Goods	Aluminium	Carbon
ACM/Bath flow from bath removal process	Primary data	Assumption n°8, assumption n°23	Assumption n°8
Partially cleaned used anodes flow from bath removal process and from butts sand cleaning process, clean butts inflow to inventory	Mass balance	Assumption n°8, assumption n°23	Mass balance
Different waste flows from butts and rod cleaning (except filter dust from rod cleaning)	Primary data	Assumption n°8, assumption n°23	Primary data
Filter dust from rod cleaning	Primary data and assumption n°5	Assumption n°8, assumption n°23	Primary data and assumption n°5
Changes in clean butts inventory	Primary data	Assumption n°8, assumption n°23	Primary data and assumption n°26
Exported clean butts	Primary data	Assumption n°8, assumption n°23	Primary data

6. *Sensitivity analysis methodology*

The table S10 shows on which parameters the sensitivity analysis was carried and to which assumption they relate to.

Table S10: List of parameters selected for the sensitivity analysis

Assumption	Parameter
A4	Carbon content of locally produced baked anode.
A5	Share of filter dust generated in rod cleaning versus anode rodding.
A9	Carbon and aluminium content of SPL1 and SPL2.
A10	Carbon content of tar waste produced in Anode Paste Plant.
A11	Carbon content of pitch volatiles burnt in anode baking furnace.
A12	Amount of SPL1 and SPL2 contained in each pot in the two smelting lines.
A18	Ratio of CO_2 to CO emitted in the anode gas and the exhaust gas from smelting.
A20	Alumina content of unsorted process waste from electrolysis.

For a given parameter *p* and a given emissions flow *E*, the sensitivities were calculated as shown *infra*.

$$E(p) = S \times p + B$$

$$S = \frac{E(p_1) - E(p_2)}{p_1 - p_2}$$

$$p\% = \frac{p+1}{p} - 1 \ (\%\ change\ of\ p\ if\ p\ is\ increased\ by\ one)$$

$$E(p)\% = \frac{E(p+1)}{E(p)} - 1$$

$$E(p)\% = \frac{E(p)+S}{E(p)} - 1 \ (\%\ change\ of\ E(p)\ if\ p\ is\ increased\ by\ one)$$

$$S\% = 0.01 \times \frac{E(p)\%}{p\%} \ (\%\ change\ of\ E(p)\ if\ p\ increases\ by\ 1\%)$$

7. *Sensitivity analysis results*

Table S11: Sensitivity of GHG emissions in smelting line 1

Slope in ton CO_2-eq per unit change in a given parameter.

Relative sensitivity in % change in emissions per % change in a given parameter.

	**Relative sensitivity in % (slope)**
Carbon content of locally produced baked anode (A4)	**0.43%** (72 965)
CO_2 to CO ratio in the smelting exhaust gas	**-0.00174%** (-0.021)
Filter dust generated in rod cleaning (A5)	**0.0008%** (161)
Carbon content of tar waste produced in Anode Paste Plant (A10)	**-0.00001%** (-3.8)
SPL-related parameters (A9, A12), aluminium content of unsorted process waste from electrolysis (A20), carbon content of pitch volatiles A11), CO_2/CO ratio in the smelting anode gas	**0%** (0)

Table S12: Sensitivity of GHG emissions in smelting line 2

Slope in ton CO_2-eq per unit change in a given parameter.

Relative sensitivity in % change in emissions per % change in a given parameter.

	**Slope / Relative sensitivity**
Carbon content of locally produced baked anode (A4)	**0.45%** (203 991)
CO_2 to CO ratio in the smelting exhaust gas	**-0.00181%** (-0.06)
Filter dust generated in rod cleaning (A5)	**0.0008%** (742)
Carbon content of tar waste produced in Anode Paste Plant (A10)	**-0.00001%** (-10.6)
SPL-related parameters (A9, A12), aluminium content of unsorted process waste from electrolysis (A20), carbon content of pitch volatiles A11), CO_2/CO ratio in the smelting anode gas	**0%** (0)

Table S13: Sensitivity of the anode baking furnace GHG emissions

Slope in ton CO_2-eq per unit change in a given parameter.

Relative sensitivity in % change in emissions per % change in a given parameter.

	**Slope / Relative sensitivity**
Carbon content of locally produced baked anode (A4)	**-8.62%** (-289 369)
Carbon content of tar waste produced in Anode Paste Plant (A10)	**0.00003%** (1.43)
Filter dust generated in rod cleaning, SPL-related parameters (A9, A12), aluminium content of unsorted process waste from electrolysis (A20), carbon content of pitch volatiles A11), CO_2/CO ratio in the smelting exhaust gas and anode gas.	**0%** (0)

8. Detailed Sankey Diagram of the anode plant subsystem

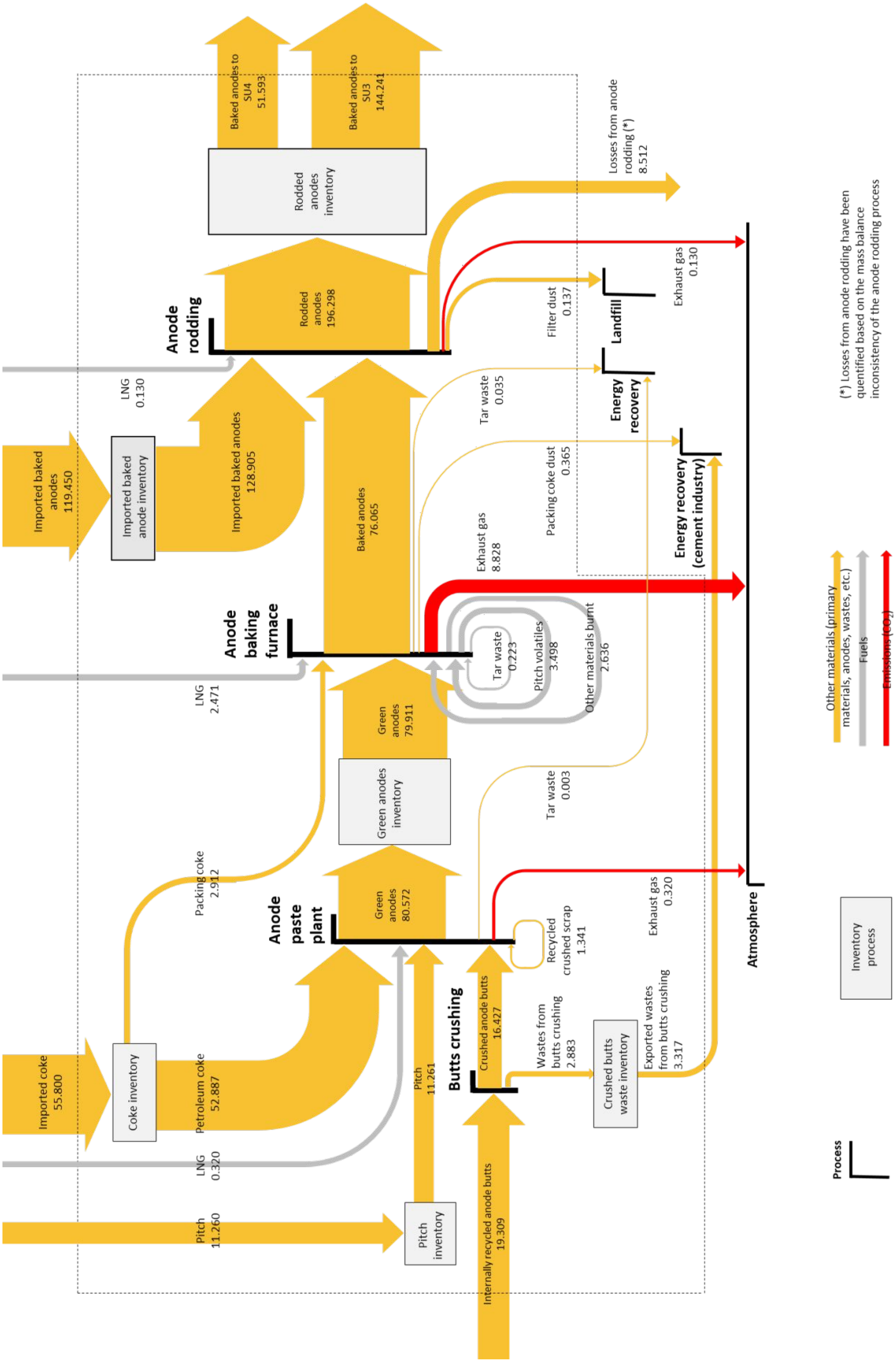

Figure S2: Detailed Sankey Diagram of the anode plant subsystem

9. *Detailed Sankey Diagrams of the anode cleaning subsystem*

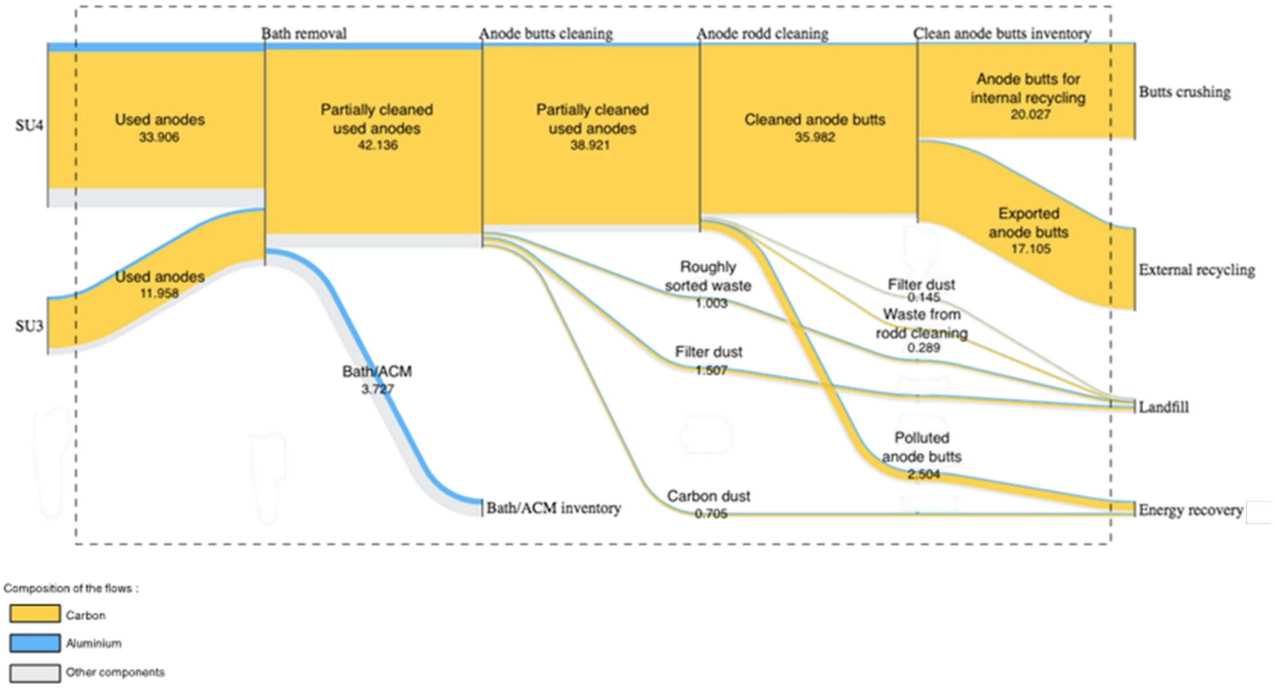

Figure S3: Anode cleaning subsystem during the year 2017 (goods layer, in kt)

Note: the process "Clean anode butts inventory" is not mass balanced. This can be explained by a stock change of this inventory during the year that is not shown on the figure.

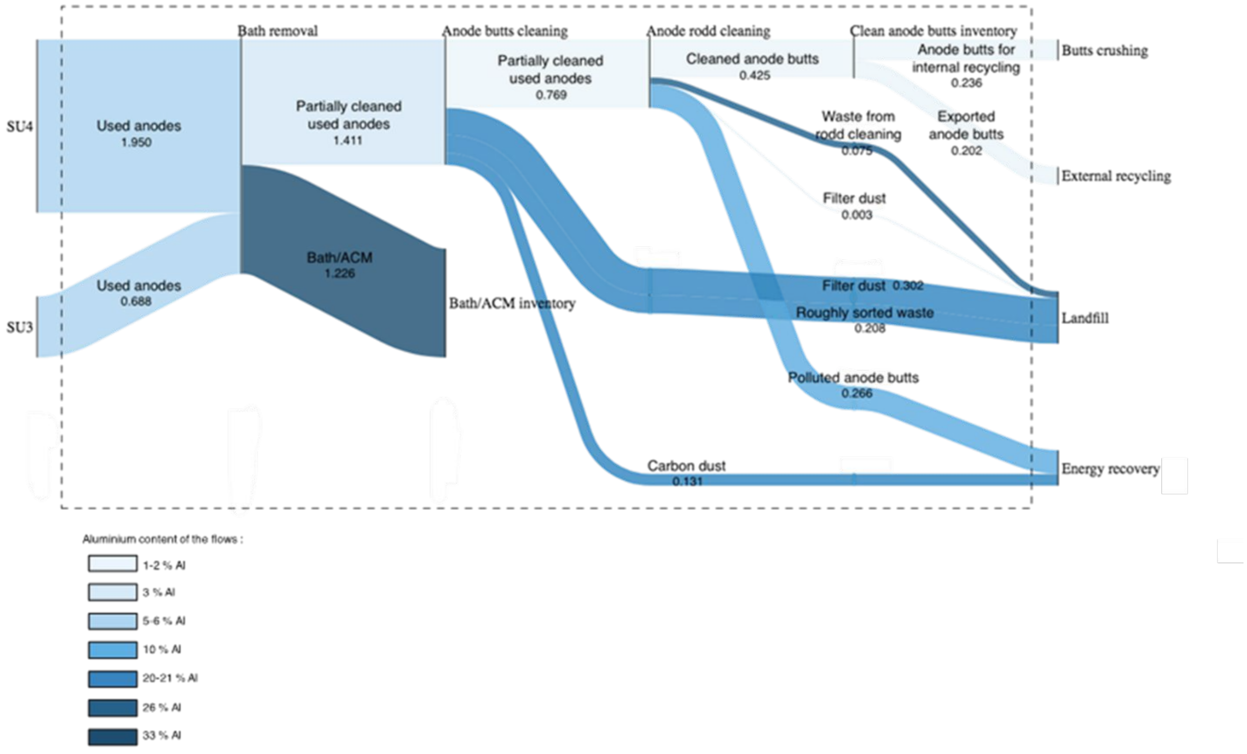

Figure S4: Anode cleaning subsystem during the year 2017 (aluminium layer, in kt)

10. Performance indicators calculations

Performance indicators in Figure 4 were calculated by dividing the real reduction reaction output/input ratios by their theoretical values that would have been obtained following the stoichiometry of an ideal alumina reduction reaction[5]:

$$\frac{1}{2} Al_2O_3 + \frac{3}{4} C = Al + \frac{3}{4} CO_2$$

Using the following standard atomic weights:

$$A_{r,\ standard}(Al) = 26.982\ ,\quad A_{r,\ standard}(O) = 15.999\ u\ ,\quad A_{r,\ standard}(C) = \ 12.011\ u$$

We obtain the following ideal theoretical ratios:

$$\begin{cases} r_{Al_2O_3,\ ideal} = \dfrac{Al\ output_{ideal}}{Al_2O_3\ input_{ideal}} = 0.529\ {}^{tAl}/_{tAl_2O_3} \\ r_{C,\ ideal} = \dfrac{Al\ output_{ideal}}{C\ input_{ideal}} = 2.995\ {}^{tAl}/_{tC} \\ r_{GHG,\ ideal} = \dfrac{Al\ output_{ideal}}{GHG\ emissions_{ideal}} = 0.817\ {}^{tAl}/_{tCO_2} \end{cases}$$

We can then compare these ideal ratios with the ones measured from the reaction that takes place in a real smelting line, where not all carbon is consumed, not all alumina is reduced and more GHG are produced, either in the form of CO, CO_2 or PFCs. Real GHG emissions were calculated using the CO_2 equivalent GWP100 provided in the AR5 of the IPCC.[6]

References

(1) Mikša, D.; Homšak, M.; Samec, N. Spent Potlining Utilisation Possibilities. *Waste Manag. Res.* **2003**, *21* (5), 467–473. https://doi.org/10.1177/0734242X0302100509.

(2) von Krüger, P. Use of Spent Potlining (SPL) in Ferro Silico Manganese Smelting. In *Light Metals 2011*; Lindsay, S. J., Ed.; Springer International Publishing: Cham, 2016; pp 275–280.

(3) Kimmerle, F. M.; Noel, L.; Pisano, J. T.; Makay, G. I.; Huglen, R. COS, CS 2 and SO 2 Emissions from Prebaked Hall Heroult Cells. In *Light metals*; TMS, 1997; pp 153–158.

(4) European Commission. Commission Regulation (EU) No 601/2012 of 21 June 2012 on the Monitoring and Reporting of Greenhouse Gas Emissions Pursuant to Directive 2003/87/EC of the European Parliament and of the Council. **2012**.

(5) Tangstad, M. *Metal Production in Norway*; Akademika Publ.: Oslo, 2013.

(6) Myhre, G.; Shindell, D.; Bréon, F.-M.; Collins, W.; Fuglestvedt, J.; Huang, J.; Koch, D.; Lamarque, J.-F.; Lee, D.; Mendoza, B.; Nakajima, T.; Robock, A.; Stephens, G.; Takemura, T.; Zhang, H. Anthropogenic and Natural Radiative Forcing. In *Climate Change 2013: The Physical Science Basis. Contribution of Working Group I to the Fifth Assessment Report of the Intergovernmental Panel on Climate Change*; Stocker, T. F., Qin, D., Plattner, G.-K., Tignor, M., Allen, S. K., Doschung, J., Nauels, A., Xia, Y., Bex, V., Midgley, P. M., Eds.; Cambridge University Press: Cambridge, UK, 2013; pp 659–740. https://doi.org/10.1017/CBO9781107415324.018.
